# Distinct pathways of adaptive evolution in *Cryptococcus neoformans* reveal a mutation in adenylyl cyclase with trade-offs for pathogenicity

**DOI:** 10.1016/j.cub.2023.08.054

**Published:** 2023-09-13

**Authors:** Zoë A. Hilbert, Joseph M. Bednarek, Mara J.W. Schwiesow, Krystal Y. Chung, Christian T. Moreau, Jessica C.S. Brown, Nels C. Elde

**Affiliations:** 1 Department of Human Genetics, University of Utah, Salt Lake City, UT 84112, USA; 2 School of Biological Sciences, University of Utah, Salt Lake City, UT 84112, USA; 3 Howard Hughes Medical Institute, University of Utah School of Medicine, Salt Lake City, UT 84112, USA; 4 Lead contact

## Abstract

Pathogenic fungi populate a wide range of environments and infect a diversity of host species. Despite this substantial biological flexibility, the impact of interactions between fungi and their hosts on the evolution of pathogenicity remains unclear. We studied how repeated interactions between the fungus *Cryptococcus neoformans* and relevant environmental and mammalian host cells—amoeba and mouse macrophages—shape the evolution of this model fungal pathogen. First, using a collection of clinical and environmental isolates of *C. neoformans*, we characterized a range of survival phenotypes for these strains when exposed to host cells of different species. We then performed serial passages of an environmentally isolated *C. neoformans* strain through either amoeba or macrophages for ~75 generations to observe how these interactions select for improved replication within hosts. In one adapted population, we identified a single point mutation in the adenylyl cyclase gene, *CAC1*, that swept to fixation and confers a strong competitive advantage for growth inside macrophages. Strikingly, this growth advantage in macrophages is inversely correlated with disease severity during mouse infections, suggesting that adaptation to specific host niches can markedly reduce the pathogenicity of these fungi. These results raise intriguing questions about the influence of cyclic AMP (cAMP) signaling on pathogenicity and highlight the role of seemingly small adaptive changes in promoting fundamental shifts in the intracellular behavior and virulence of these important human pathogens.

## In brief

Interactions between environmental microbes, like many fungi, and diverse hosts can fundamentally alter the biology and pathogenicity of these organisms. Using natural genetic variation and laboratory-based evolution, Hilbert et al. reveal the tempo, mode, and mechanisms by which adaptive evolution occurs in a clinically relevant fungal pathogen.

## INTRODUCTION

The ability to thrive in different environments is critical for the evolutionary success of most organisms. For pathogenic microbes, adaptive traits that facilitate survival within host cells or tissues evolve through repeated interactions with host immune factors or exposure to host-like conditions. However, for opportunistic environmental pathogens, including many pathogenic fungi, the mechanisms by which these organisms gain the ability to infect mammalian hosts are not well understood. Furthermore, our understanding of how fungi adapt over short timescales within animal hosts in response to pressures from host immune cells, such as macrophages, is similarly limited.

The human fungal pathogen *Cryptococcus neoformans* is a globally distributed haploid yeast ubiquitous in the environment and often found associated with trees or bird guano.^1^ Human infection with *C. neoformans* occurs primarily through inhalation of fungal spores or desiccated yeasts into the lungs where, in immunocompetent patients, infection is either cleared or contained in a persistent state.^2^ Symptomatic disease is observed mostly in immunocompromised patients, where dissemination to the brain leads to cryptococcal meningitis, which is lethal unless treated.^3^ Although *C. neoformans* is an effective and important human pathogen, the evolutionary pressures and genomic adaptations that led to the emergence of pathogenicity in this species remain elusive.

Some evolutionary studies of virulence in *C. neoformans* focused on the characterization of large collections of isolates obtained from both clinical and environmental sampling.^4–10^ Genome-wide association studies identified genomic changes that may underlie the increased success of some of these strains as pathogens.^4,9,10^ Although such studies are critical in identifying key genes and pathways associated with increased pathogenicity, they are less useful in identifying selective pressures driving acquisition of these traits. Instead, complementary studies have focused on the role of environmental hosts, such as amoebae and nematodes, in shaping the evolution of virulence in *Cryptococcus* species.^11,12^

Amoebae are well studied as a potential source of environmental selective pressure for diverse microbes, given similarities to macrophages of animal immune systems. Like macrophages, amoebae can phagocytose microorganisms and undergo many similar processes following phagocytosis.^13^ Studies of bacterial species, like *Legionella pneumophila*, identified virulence factors required for success in both amoeba species and mammalian hosts, providing strong evidence that similarities across host-cell environments could select for broadly effective virulence strategies.^14–17^ For *C. neoformans*, the polysaccharide capsule has been shown to have protective roles against both amoeba and mammalian hosts, and other features of the intracellular behavior of *C. neoformans* appear conserved across these evolutionarily diverse species.^18–21^ However, whether pathogenicity can be evolved through repeated interactions with amoebae or other environmental hosts and the mechanisms through which this occurs have not been well studied.

Adaptation within mammalian hosts also has the potential to drive selection within fungal populations with important implications for disease progression. “Microevolution” studies performed by analyzing serial samples from human patients revealed numerous adaptive phenotypic changes in these strains over long time periods, although correlation between genetic changes and adaptive phenotypes has proven challenging to assess.^22–24^ Serial *in vitro* passaging schemes, on the other hand, provide a powerful system to identify phenotypic changes involved in host adaptation *and* identify the molecular mechanisms associated with such changes.^25–28^ Few studies, though, have compared the adaptive changes that occur in response to exposure to different host species. In addition, most serial passaging experiments focus on changes that occur in commonly used laboratory strains or closely related clinical isolates, which may not capture the evolutionary trajectories taken by divergent isolates of these fungal species.

Here, we used clinical and environmental isolates of *C. neoformans* to identify strain-level variation in survival in either environmental (amoeba) or mammalian (mouse macrophage) hosts. We serially passaged one of these environmental isolates to isolate host-adapted strains with enhanced abilities to survive in the intracellular niches of highly diverged host organisms. Remarkably, this approach revealed a single missense mutation in the *CAC1* adenylyl cyclase gene sufficient to confer enhanced macrophage replication, which modulates the balance between host adaptation and pathogenicity in this important human fungal pathogen.

## RESULTS

### Natural variation in *C. neoformans* strain replication in amoebae and macrophages

**Figure 1.**
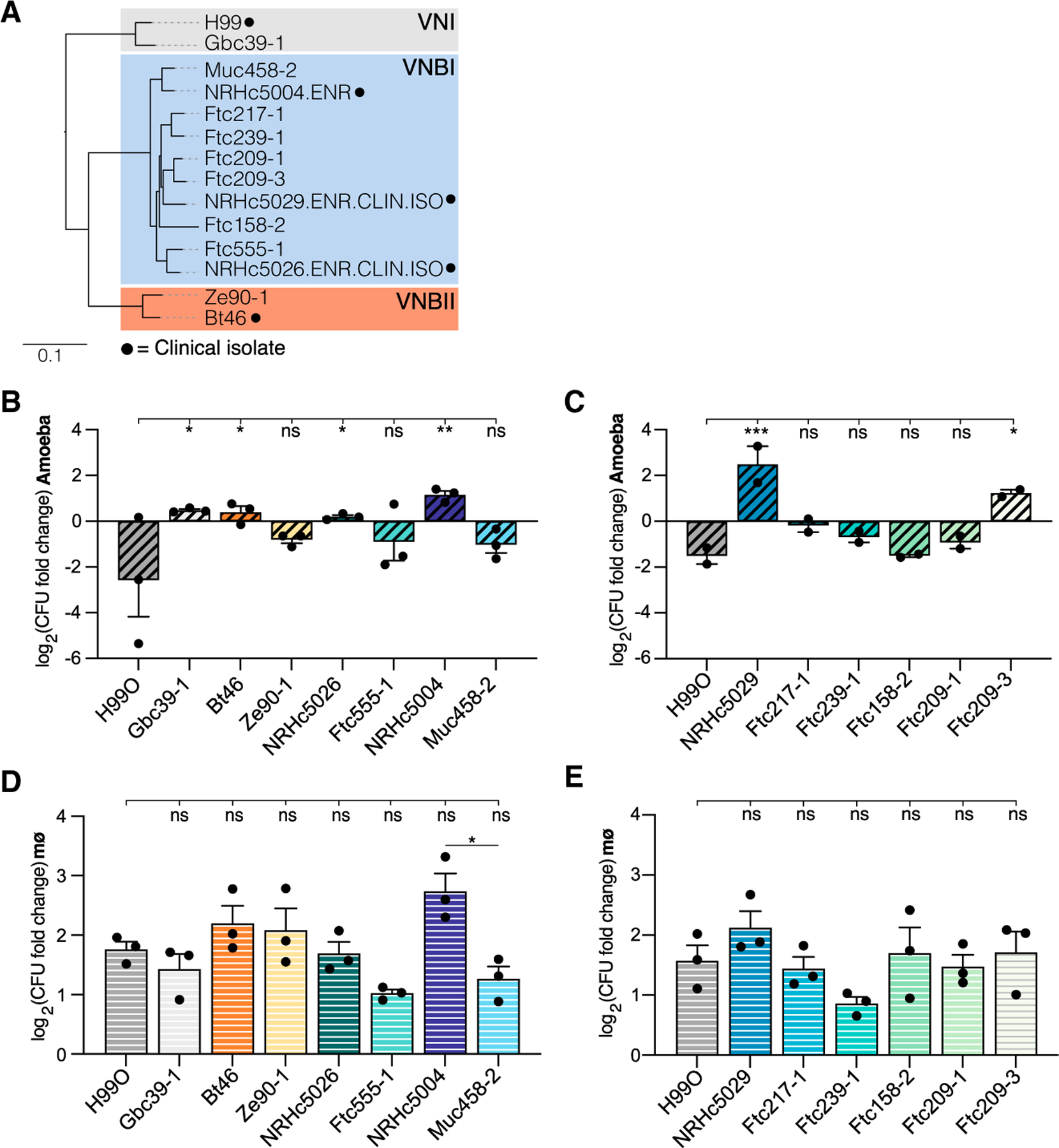
Phenotypic variation of *C. neoformans* clinical and environmental isolate survival in amoebae or macrophage hosts (A) Phylogenetic tree of clinical and environmental *C. neoformans* strains used in this paper. Tree was extracted from a larger published phylogeny using the R package Treehouse.^4,29^ Lineages are indicated by colored boxes and labels, and black dots indicate clinical isolates. All other strains were isolated from environmental sources. Scale bar, 0.1 substitutions/site. (B and C) Replication of *C. neoformans* strains in co-incubation experiments with *A. castellanii*. (D and E) Replication of the same strains in co-incubation experiments with J774A.1 macrophage cells. For (B)–(E), bars show the average values ± SEM across 2–3 independent experiments on different days. Dots indicate the average values for 3 replicates for each independent experiment. Significance was assessed by comparison to the H99O reference strain by ordinary one-way ANOVA followed by Dunnett’s multiple comparisons test. Pairwise comparison of NRHc5004 and Muc458-2 in (D) was assessed by unpaired t test with Welch’s correction. *p < 0.05; **p < 0.01; ***p < 0.0001; ns, not significant. See also Figures S1 and S5.

We compared the replication of 14 unique *C. neoformans* isolates in cells of the amoeba, *Acanthamoeba castellanii*, or in the J774A.1 mouse macrophage-like cell line (Figure 1). The strains represent three of the four major non-recombining *C. neoformans* sub-lineages—VNI, VNBI, and VNBII—and are predominantly environmental isolates, although several clinical isolates were also included (Figure 1A).^4^

When incubated with *A. castellanii* for 24 h, we observed robust killing of cryptococcal cells for nearly all strains tested, despite roughly similar growth in the absence of amoebae and comparable phagocytosis rates (Figures 1B, 1C, S1A, and S1B). Notably, we identified two strains with enhanced replication in amoebae—NRHc5004 and NRHc5029—both of which were isolated from clinical sources. In contrast, all *C. neoformans* isolates tested were able to replicate to some extent when incubated with mouse macrophages (Figures 1D and 1E). The clinical isolate NRHc5004 also exhibits enhanced replication in macrophages compared with other closely related strains, underscoring the possibility that there are strategies for intracellular replication that confer success across evolutionarily diverse hosts. Again, we observed no significant differences in phagocytosis by macrophages, suggesting that differences in intracellular replication are primarily responsible for the strain variation we observe in these experiments (Figures S1C and S1D).

In addition to strain-specific variation in macrophage replication, we noted even more variable growth of these strains under tissue culture conditions in the absence of macrophages (Figures S1E and S1F). Intriguingly, the environmental isolate Ftc555-1 was killed by incubation in tissue culture media alone, but growth could be partially rescued through the addition of macrophage cells to the culture. We reasoned that we could exploit the preference of Ftc555-1 for the macrophage intracellular environment for serial passaging and host adaptation studies without excessive growth outside of these cells.

### Serial passaging selects for host-adapted strains

**Figure 2.**
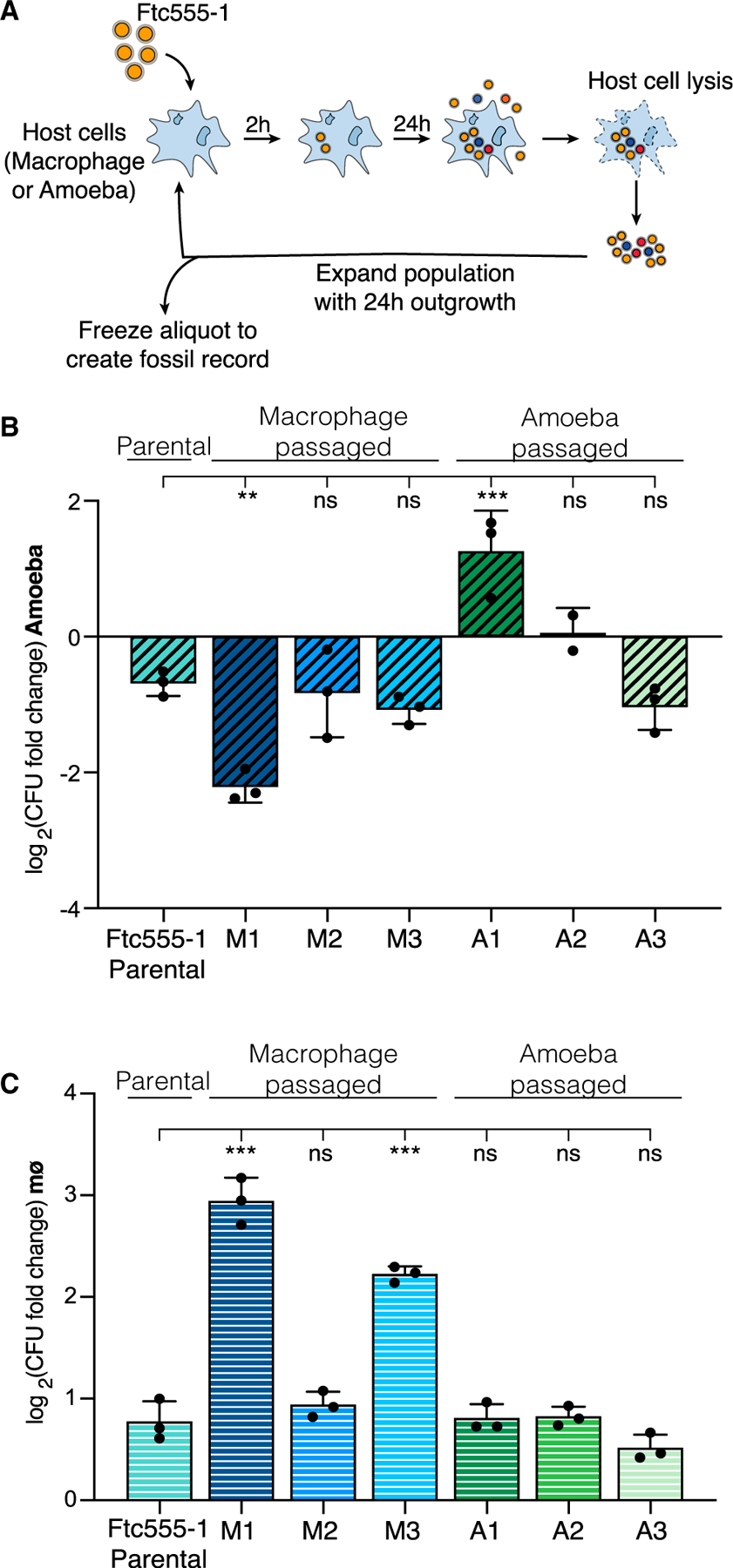
Serial passaging of *C. neoformans* through macrophages or amoeba cells selects for host-adapted strains (A) Schematic of the serial-passaging setup used in this study. See also STAR Methods. (B) Replication of passaged strains in co-culture with *A. castellanii*. Single colonies from each individually evolved population were tested for growth in amoeba. Strains M1–M3 were passaged through macrophages; A1–A3 were passaged through amoeba. (C) Replication of passaged strains in co-culture with mouse macrophage-like J774A.1 cells. The same single colonies were tested as in (B), and data are plotted identically. For (B) and (C), plotted data are from one representative experiment. Error bars indicate SD and each dot indicates the value for one biological replicate from that experiment. Significance was assessed between the parental strain values and all evolved strains by ordinary one-way ANOVA followed by Dunnett’s multiple comparisons test. **p < 0.01; ***p < 0.0001; ns, not significant. See also Figure S2.

We devised a serial-passaging scheme where Ftc555-1 was repeatedly subjected to phagocytosis by either *A. castellanii* amoebae or J774A.1 macrophages (Figure 2A; STAR Methods). Three independent populations were evolved in parallel in each cell type and are hereafter referred to as lines M1–M3 (for macrophage-passaged) and A1–A3 (for amoeba-passaged). We ran courses of experimental evolution for a total of 12 passages over the span of roughly 1 month of continuous culturing. At the end of the experiment, we collected each population and froze down pooled cultures. Importantly, aliquots of each passage were frozen over the course of the experiment to create a “fossil record,” which allowed for high-resolution tracking of adaptive mutations over time and assessment of intermediate phenotypes.

Following the final passage, we analyzed the ability of individual colonies from each population to replicate in either amoebae or macrophage hosts (Figures 2B and 2C). In amoebae, only the A1 population showed significant increases in replication (Figure 2B). Enhanced replication of this strain is specific to growth in culture with host cells, as media-only replication rates remained unchanged from the parental strain (Figure S2C). In macrophages, we observed enhanced replication in two of the three macrophage-passaged isolates (Figure 2C). Notably, in both macrophage-adapted strains (M1 and M3), we also observed a marked improvement in growth under tissue culture conditions in the absence of macrophages (Figure S2C). Importantly, there were minimal differences in phagocytosis of these strains compared with the parental strain, indicating that the phenotypes we observe are independent of changes in phagocytosis (Figures S2A and S2B).

### A macrophage-evolved strain outcompetes the parental for growth in macrophages

Of the strains recovered from our serial-passaging approach, the M1 strain stood out with a nearly 5-fold increase in macrophage replication compared with the parental strain (Figure 2C).

We sought to determine the relative fitness advantage of this M1 isolate compared with parental Ftc555-1 through competition experiments in which we co-incubated a nourseothricin-resistant (NAT^R^) version of the parental strain with unlabeled strains of interest and assessed growth in macrophages. Differential plating of the recovered populations from these experiments allowed us to determine the relative growth advantage of each strain.

**Figure 3.**
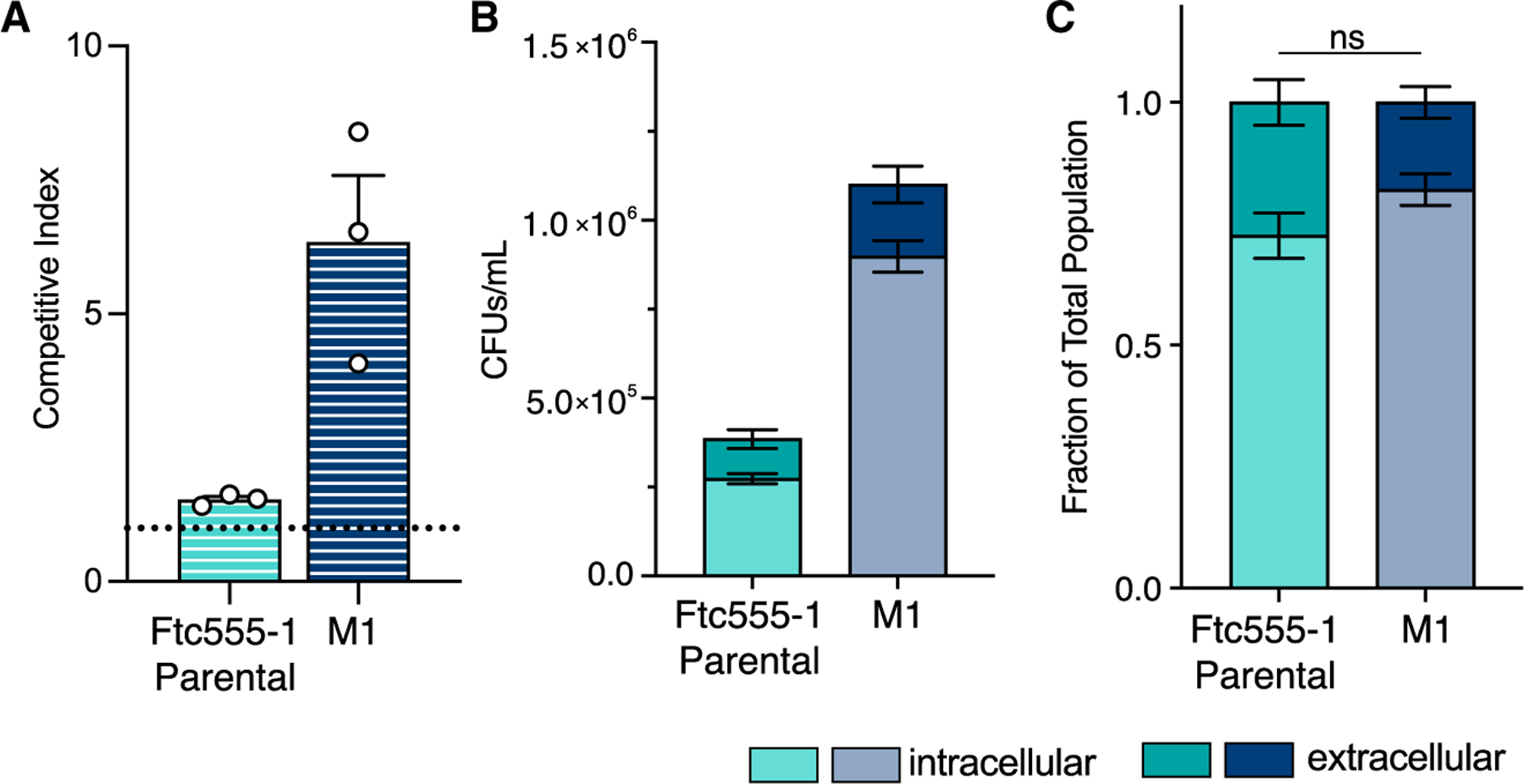
The evolved M1 strain outcompetes the parental strain in macrophages (A) Competition experiments between the NAT^R^-labeled parental Ftc555-1 and unlabeled parental (teal), and M1 (navy blue) strains in macrophage cells. Competitive indices (CIs) are calculated as indicated in STAR Methods. A CI = 1 (dotted line) indicates equal growth of the two strains. Plotted values indicate the average value ± SEM of three replicate experiments carried out on three separate days; dots indicate the average value of replicates from a single experiment. (B) Total CFU counts of Ftc555-1 and M1 strains following macrophage co-incubation. Intracellular (light shades) and extracellular (dark shades) populations were collected and plated separately. Bars show the average values ± SEM from two independent experiments on separate days. (C) Intracellular and extracellular growth following macrophage co-incubation plotted as a fraction of the total population. The same data from (B) but presented as a fraction rather than as raw CFU counts. Significance was assessed by unpaired t test with Welch’s correction. ns, not significant. See also Figure S3.

The M1 strain outcompeted the NAT^R^ parental by over 5-fold in macrophages (Figure 3A). In contrast, when the parental Ftc555-1 was instead competed against the NAT^R^-labeled version of itself as a control, we observed a competitive index of ~1. Therefore, the evolved M1 isolate has a strong fitness advantage over parental Ftc555-1 for growth in the macrophage environment.

In addition to revealing the fitness advantage of the M1 strain in macrophages, our competition experiments revealed that M1 was able to outcompete the parental Ftc555-1 strain by nearly 40-fold in tissue culture media alone (Figure S3A). To address the possibility that increased macrophage replication in M1 reflects enhanced extracellular growth following escape from macrophages, either through killing of the host cell or non-lytic exocytosis, we assessed the role of extracellular growth in contributing to the M1 phenotype through several methods.^30–32^

First, we determined the amount of extracellular growth that occurs under normal experimental conditions. We performed co-incubation experiments with macrophages where we separated intracellular and extracellular populations and enumerated colony-forming units (CFUs) from each sub-population. The M1 CFUs were significantly higher than the ancestral strain for both the intracellular and extracellular populations (Figure 3B). However, when assessed as a fraction of the total recovered CFUs, there was no significant difference in the percentage of the population that was extracellular at the end of the experiment (Figure 3C). This demonstrates that the M1 strain is not escaping macrophages at a noticeably higher rate than the parental strain, and extracellular replication is not a confounding issue.

We also assessed extracellular growth differences by using the antifungal drug fluconazole to limit *C. neoformans* replication in media (Figures S3B–S3D). Following phagocytosis by macrophages, *Cryptococcus* cells are somewhat protected from the effects of fluconazole, which allowed us to perform competition experiments where replication was limited to the macrophage intracellular environment (Figures S3C and S3D). The M1 strain had a nearly identical competitive index (>5-fold advantage) when grown in the presence *or* absence of fluconazole (Figures 3A and S3D), demonstrating that the fitness advantage in M1 is due to enhanced replication in the intracellular niche. Although we observed no difference in the minimum inhibitory concentration (MIC) for fluconazole between the two strains, the evolved M1 strain maintained a modest fitness advantage in media with fluconazole present (Figures S3B and S3D). Combined, these data demonstrate that experimental evolution selected for a strong intracellular fitness advantage in the M1 macrophage-passaged strain.

### Enhanced growth of M1 is caused by a single-nucleotide polymorphism in the adenylyl cylase gene

**Figure 4.**
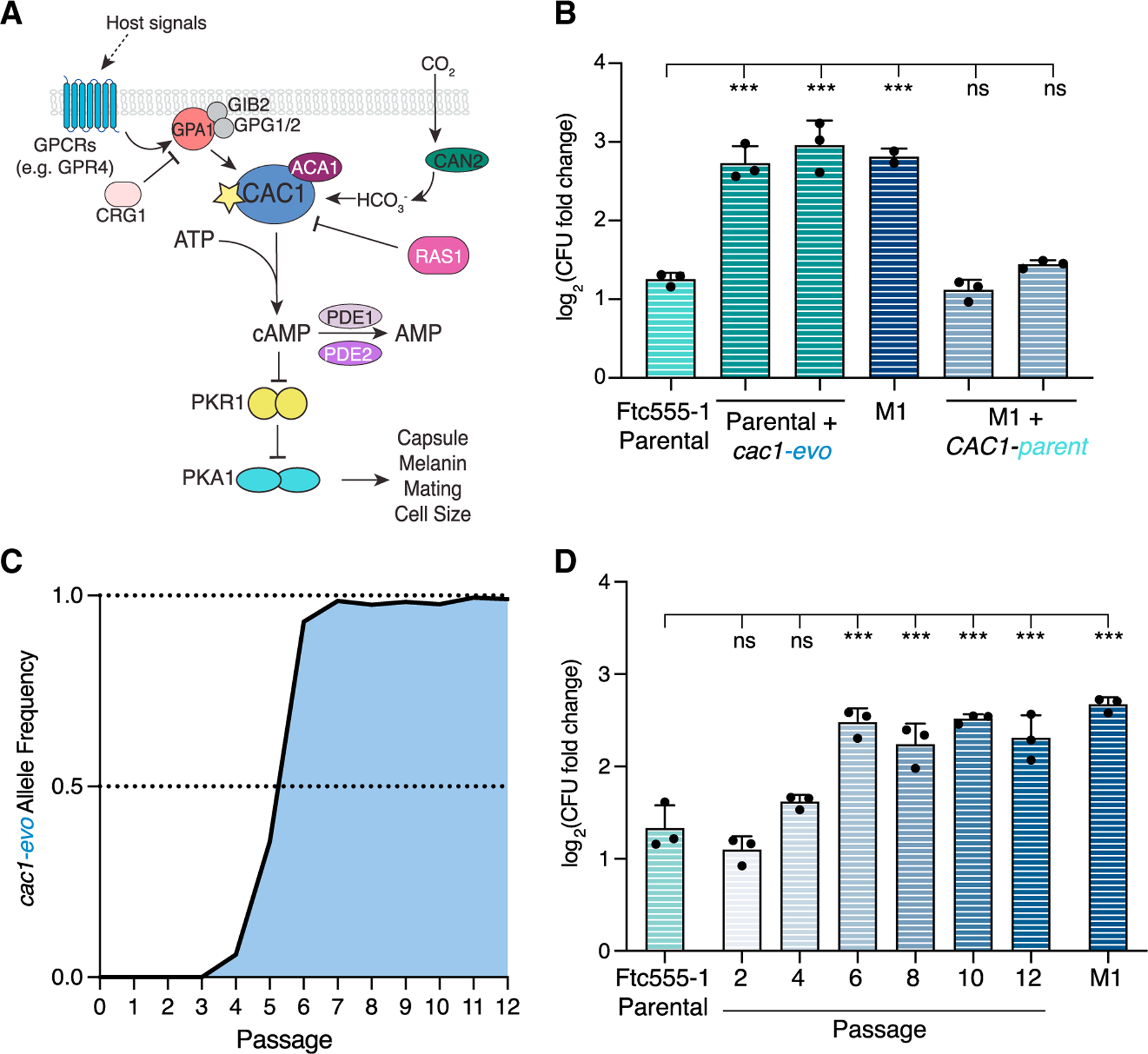
A single-nucleotide change in the CAC1 adenylyl cyclase gene is necessary and sufficient for the enhanced growth of M1 (A) Schematic of the cAMP signaling pathway in *C. neoformans* as worked out in the KN99 genetic background. *CAC1*, indicated with the yellow star, regulates the production of cAMP downstream of a number of host signals including nutrients and carbon dioxide. Production of cAMP leads to the activation of the Pka1 protein kinase, which functions to regulate many key aspects of *C. neoformans* biology and virulence, including capsule and melanin production, mating, titan cell formation, and others. Activation of Cac1 and modulation of cAMP levels is achieved through the interactions with a number of other genes, as indicated. (B) Replication phenotypes of allele-swap strains in macrophage co-culture experiments. Allele-swap samples tested are two independently derived strains from CRISPR-mediated allele-swap transformations (STAR Methods). (C) Allele frequency of the *cac1-evo* allele over the course of the evolution experiment. The number of reads containing the R1227P mutation in sequencing data from each passage population were counted and normalized to the read depth to calculate allele frequencies. (D) Growth of intermediate passage populations in co-incubation experiments with macrophages. Each tested sample represents a non-homogeneous population of cells from the indicated passage. For (B) and (D), data shown are the average value ± SD from one representative experiment with each dot representing a single replicate from that experiment. Significance in these experiments was assessed compared with the parental strain by ordinary one-way ANOVA followed by Dunnett’s multiple comparisons test. ***p < 0.0001; ns, not significant. See also Figure S4.

To identify the genetic changes underlying the adaptive phenotype of the M1 strain, we used a combination of Nanopore and Illumina sequencing to assemble a high-quality reference genome of the Ftc555-1 starting isolate. We then performed whole-genome sequencing on several individual colonies from the M1 population and on a pooled culture from the final passage of our evolution experiment. Surprisingly, we found only a single-nucleotide change differentiating the M1 strain from the originating Ftc555-1 isolate. This mutation, which was fixed in both single colonies and the pooled culture, changes a single amino acid—Arg1227-Pro—in the gene *CAC1* (CNAG_03202). *CAC1* encodes the adenylyl cyclase gene in *C. neoformans*, which is required to produce cyclic adenosine monophosphate (cAMP) and for the regulation of numerous aspects of *Cryptococcus* biology (Figure 4A).^33–37^

The importance of *CAC1* for *C. neoformans* biology combined with the lack of other mutations in the M1 strain strongly suggested that the Arg1227Pro mutation was causative for the adaptive phenotypes in this strain. We swapped this single nucleotide in both the parental Ftc555-1 and M1 strains using CRISPR-mediated gene editing and tested allele-swapped strains for growth in our macrophage co-culture experiments (Figure 4B). When the evolved *CAC1* allele—referred to from here on as *cac1-evo*—was introduced into the parental Ftc555-1 background, this was sufficient to cause a striking increase in replication in macrophage cells as well as in tissue culture media alone (Figures 4B and S4A). In the reciprocal swap, where the *cac1-evo* allele was replaced with the parental version (*CAC1-parent*), we observed a complete loss of the enhanced growth phenotypes. These data indicate that this single point mutation in the *CAC1* adenylyl cyclase gene is both necessary and sufficient to confer the drastically altered macrophage growth phenotype that defines our evolved M1 strain.

### Evolutionary dynamics of the *CAC1* polymorphism reveal a strong fitness advantage for the evolved allele

We were curious about the emergence and dynamics of the *cac1-evo* allele over this short course of evolution. Taking advantage of the fossil record created during the experiment, we sequenced all intermediate passage populations and analyzed the *CAC1* locus. By passage four, a very low number of reads (ten total reads, allele frequency = ~5%) contained the evolved allele, suggesting that the mutation arose *de novo* around this time (Figure 4C). The *cac1-evo* allele frequency rose quickly over subsequent passages: greater than 90% of reads carried the mutation at passage six, and it had fully swept through the population by passage seven. This suggests that the *cac1-evo* allele confers a strong fitness advantage, in alignment with the competitive advantage we had observed in earlier experiments (Figure 3).

To examine the correlation between the *cac1-evo* allele and adaptation to macrophage host cells, we compared the growth of intermediate passage populations in macrophage co-incubation experiments (Figures 4D and S4B). In agreement with the sequencing data, we see no significant difference in growth of the early passage populations. By passage four, replication in macrophages trended upward and by passage six was indistinguishable from the clonal M1 strain.

We also assessed the competitive advantage of each passage population in head-to-head competition experiments with the parental strain (Figures S4C and S4D). We noted an increase in the competitive index for growth in macrophages beginning around passage six and reaching maximal levels at roughly passage 8, consistent with individual population tests (Figure S4C). Media-only competition experiments revealed a marked increase in competitive advantage beginning in passage five, which remained largely unchanged across subsequent passage populations (Figure S4D). Together, these results link the emergence of macrophage adaptation in the M1 strain to the mutation of *CAC1* and highlight the strength of the fitness advantage conferred by the *cac1-evo* allele, which rapidly swept through the population.

### The *cac1-evo* allele causes phenotypic changes in pathogenicity-associated traits

**Figure 5.**
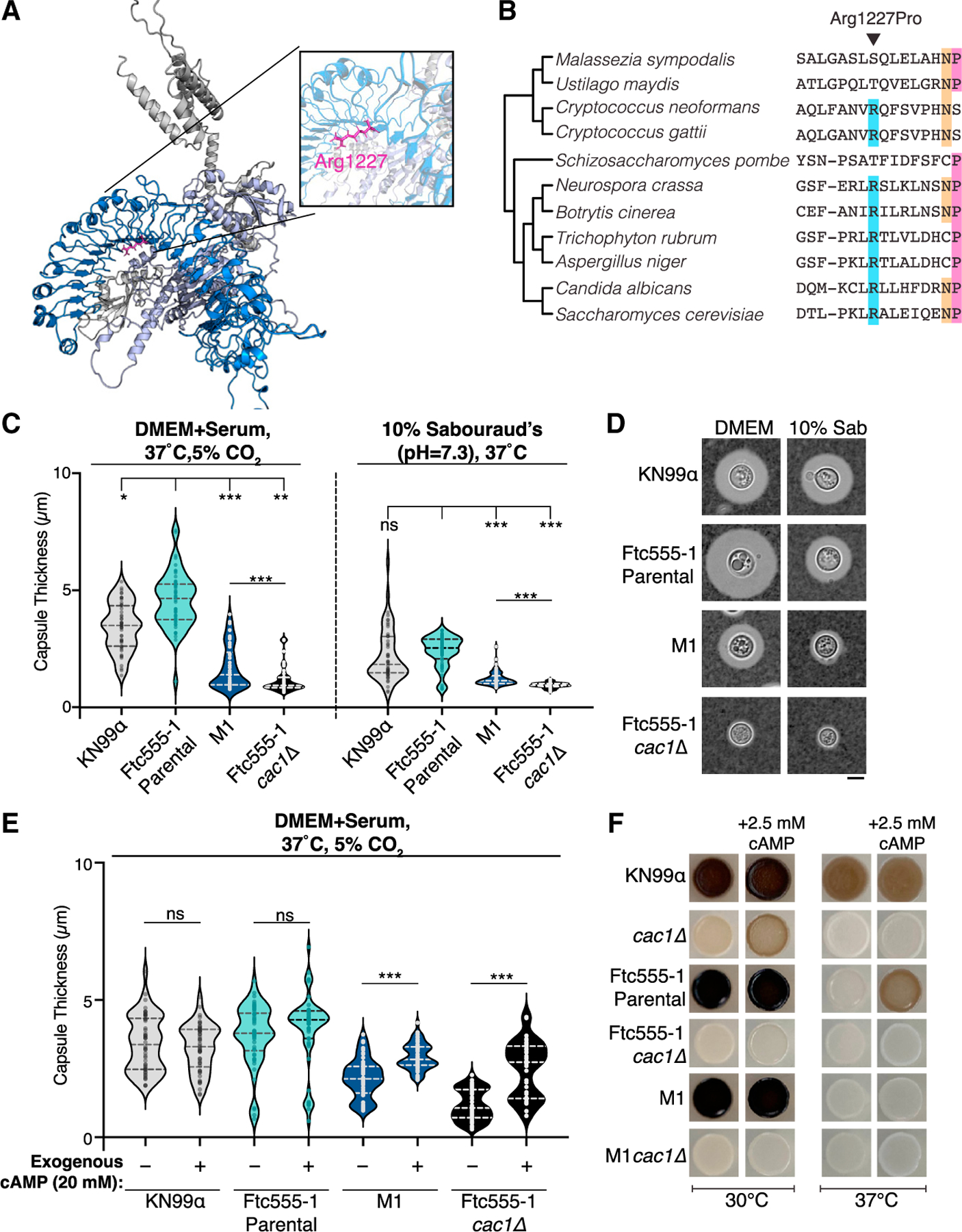
The R1227P mutation falls into the LRR domain of Cac1 and affects pathogenicity-associated traits of C. neoformans (A) AlphaFold2 generated structural model of the Ftc555-1 parental Cac1. The catalytic domain is indicated in light purple shading, LRR indicated by blue shading, and other protein regions are shown in gray. Inset shows the position of the mutation in the *cac1-evo* allele (pink). (B) An arginine at amino acid position 1227 is conserved across fungi. Sequence alignments of adenylyl cyclase orthologs from a panel of fungal species; only the region surrounding the isolated mutation is shown. Position 1227, the site of the *cac1-evo* mutation, is indicated by the black arrow, and species with the conserved arginine at this site are shaded in blue. Other residues conserved among fungi are indicated with colored shading toward the 3′ end of this region. (C) Measurements of capsule size under two different inducing conditions: tissue culture conditions (left side) and 10% Sab’s (right side). (D) Representative images of cells from (C). All images are at the same scale. Scale bar, 5 μm. (E) Capsule size measurements under tissue culture conditions with or without the addition of 20 mM exogenous cAMP. (F) Melanization of all strains at 30°C (left) and 37°C (right) on L-DOPA plates with or without 2.5 mM cAMP. The dark color of the yeast indicates melanization. In (C) and (E), violin plots show the distribution of measurements for 50 cells from one representative experiment. For all violin plots, dotted lines indicate the median and quartiles. For (C), significance was assessed compared with the Ftc555-1 parental strain via Kruskal-Wallis test with Dunn’s multiple comparisons test. Significance between the M1 and Ftc555-1 *cac1Δ* in (C) and all comparisons in (E) were determined by Mann-Whitney test. *p < 0.05; ***p < 0.0001; ns, not significant. See also Figure S5.

cAMP signaling downstream of Cac1 regulates several key pathways important for the pathogenicity of *C. neoformans*.^38^ Paired with the high fitness consequences associated with the *cac1-evo* allele, this raises the question of how the Arg1227Pro mutation affects cryptococcal biology to promote replication in macrophages. Structural modeling of the Ftc555-1 Cac1 protein using AlphaFold revealed that the Arg1227Pro mutation falls outside of the catalytic domain and sits at the edge of the concave surface of the leucine-rich repeat (LRR) domain (Figure 5A). Although little is currently known about this region of the LRR, this domain is a conserved feature of all fungal adenylyl cyclases and has been shown in other species to mediate interactions with regulatory proteins such as Ras.^39–41^

Interestingly, sequence comparisons of the adenylyl cyclase orthologs of other pathogenic and nonpathogenic fungi revealed conservation of an arginine at the corresponding amino acid position in most fungal species examined, despite overall low sequence conservation (Figure 5B). Tellingly, in species where arginine is not conserved, we see no evidence for substitutions as potentially disruptive as a proline at this site. Furthermore, we looked for variation at this position across the >500 clinical and environmental isolates of *C. neoformans* with sequence information available within public databases. We saw 100% conservation of the arginine at amino acid position 1227 across all *CAC1* sequences in these isolates, although there was coding sequence variation in other regions of the gene (Figure S5A). Together, the conservation of this site within *Cryptococcus* and across fungi is highly suggestive that it is functionally important for the activity of Cac1.

We next probed the impact of this mutation through a series of assays analyzing its effects on downstream pathogenicity-associated phenotypes, including capsule production and melanization. Both traits have previously been shown to require cAMP signaling and play important roles during infection.^33,42^ Capsule is induced *in vitro* by specific culturing conditions that mimic host-like environments.^43–45^ We tested the ability of our strains to induce capsule using two different induction protocols (STAR Methods); mutants in the cAMP signaling pathway can be sensitive to specific capsule induction conditions, motivating our decision to test multiple protocols.^44^ We observed both strain-specific and condition-specific differences in capsule induction in the Ftc555-1 parental and M1 evolved strains (Figures 5C and 5D). Cells from the parental strain produced significantly larger capsules than M1 cells, whereas capsule sizes were roughly comparable between Ftc555-1 parental cells and those of the laboratory strain, KN99α. M1 cells produced limited capsule under tissue culture conditions (Figure 5C, left). However, capsule production was markedly reduced in M1 cells incubated in the Sab’s inducing media (Figures 5C and 5D, right).

A *cac1Δ* mutant in the Ftc555-1 background produced very little capsule in either condition, consistent with Cac1 activity being required for capsule production in the Ftc555-1 background, as it is in reference strains.^33^ Additional genetic analysis of upstream genes in the cAMP pathway, *GPA1* and *ACA1*, revealed conserved roles in the regulation of cAMP and capsule induction in the Ftc555-1 background (Figure S5B). In contrast, deletion of the G protein-coupled receptor (GPCR), *GPR4*, which is required in reference strains for capsule induction upstream of Cac1 activity, had no effect in the Ftc555-1 background, regardless of *CAC1* allele.^46^ This shows that cAMP signaling is altered in Ftc555-1-derived strains with potentially fundamental impacts on how environmental inputs are transduced by GPCRs to influence Cac1 activation.

To further probe the nature of the *cac1-evo* allele in M1, we asked whether exogenous cAMP added to the media could rescue defects in capsule size that we observed in these experiments (Figures 5E, S5C, and S5D). Addition of high levels (20 mM) of cAMP partially rescued capsule production of both the M1 strain and the *cac1Δ* mutant in the Ftc555-1 background (Figures 5E and S5C). However, exogenous cAMP did not fully rescue the phenotypes of these strains, in contrast to the KN99α *cac1* deletion that exhibits a robust rescue of capsule defects when supplemental cAMP is provided (Figure S5D).

In addition to capsule, we assessed melanization phenotypes of our strains and observed no differences across the M1, Ftc555-1 parental, and KN99α strains at 30°C (Figure 5F, left). Because the ability to melanize is preserved in strains carrying the *cac1-evo* allele but abolished in strains with *CAC1* deletions, this confirms that the Arg1227Pro mutation does not confer a complete loss of function of Cac1 activity. Surprisingly, when we tested melanization at 37°C, both the Ftc555-1 parental and M1 strains had severe melanization defects (Figure 5F, right). Addition of exogenous cAMP was sufficient to rescue the defect of the parental strain at 37°C but had no effect on melanization of M1 or the *cac1Δ* mutants in any genetic background. At 30°C, exogenous cAMP differentially affected melanization across the *cac1Δ* mutants in different genetic backgrounds.

Although these data suggest *cac1-evo* represents a partial loss-of-function allele, the varying effects of this allele on different traits, across differing conditions and with varying responsiveness to cAMP supplementation reveals hidden complexity in the regulation of essential pathogenicity-associated traits. Collectively, these results point to different activity requirements and/or regulatory partners for the Cac1 pathway under distinct nutritional and environmental conditions among different strains.

### *CAC1* mutation restricts cell size modulation

We next tested the impact of the *cac1-evo* allele on modulation of cell size. The ability of *C. neoformans* to undergo morphological shifts and generate heterogeneously sized populations plays an important role in the success of *in vivo* infections. The formation of large polyploid titan cells (>10 μm diameter) allows for evasion of phagocytosis by immune cells and is crucial for establishing infection in the lungs; on the other end of the size spectrum, the recently described small seed cells facilitate dissemination of *C. neoformans* to extrapulmonary sites.^37,47,48^ cAMP signaling has previously been implicated in titan cell formation, but whether this pathway plays a general role in controlling all morphological shifts is unknown.

**Figure 6.**
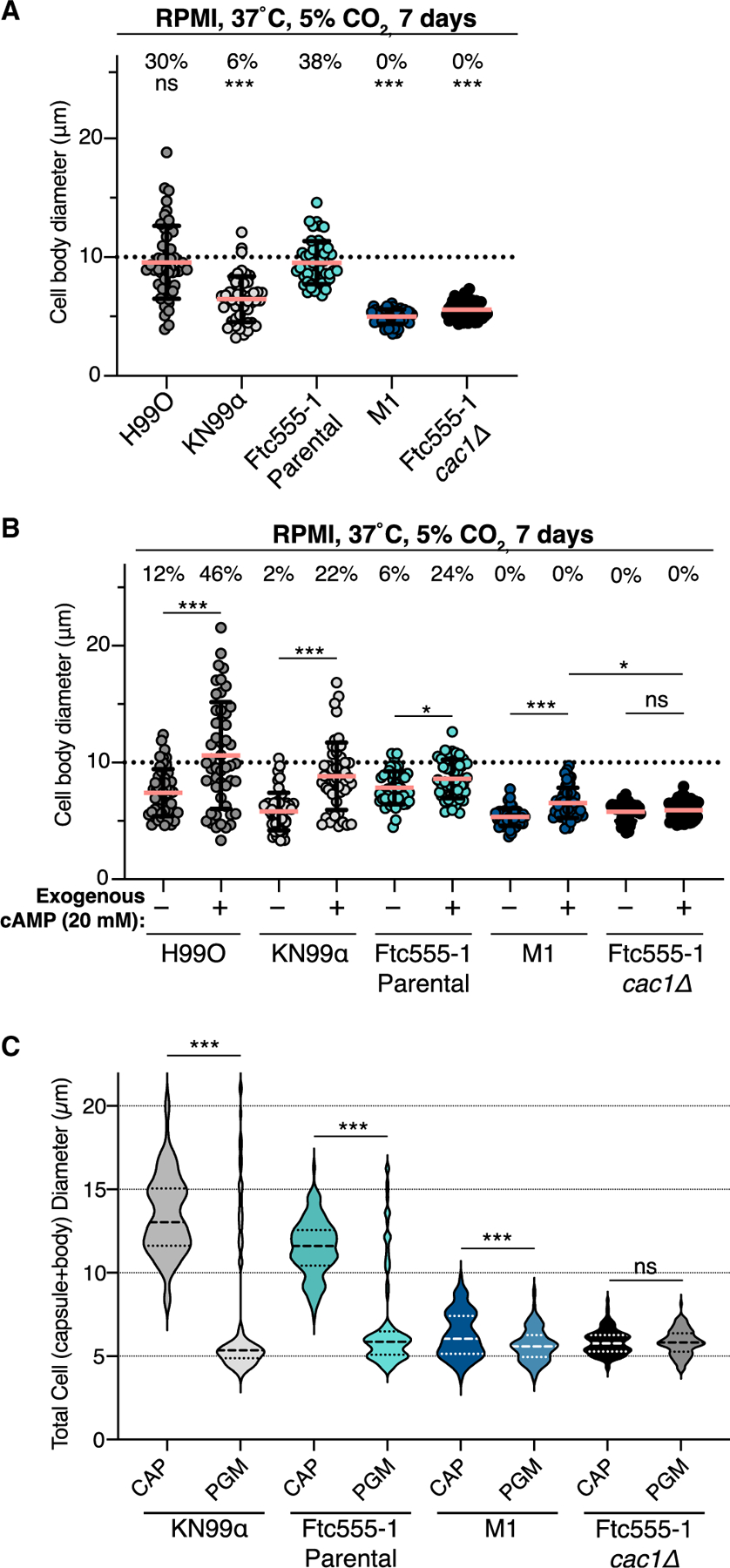
CAC1 mutations impact the ability of cells to modulate size (A and B) Measurements of cell body diameter from cells grown under titan-cell-inducing conditions (RPMI for 7 days) without cAMP supplementation (A) or with 20 mM cAMP added to the media (B). 50 cells were measured per strain. Percentages above each strain/condition indicates the percent of cells with a diameter >10 μm (indicated by the dotted line). Error bars show mean ± SD for one representative experiment. (C) Total cell diameter of CAP-grown cells after exposure to PGM. Violin plots show measurements for >100 cells from one representative experiment. For (A), significance was assessed compared with the Ftc555-1 parental strain via Kruskal-Wallis test with Dunn’s multiple comparisons test. For (B) and (C), pairwise significance between matched strains under different conditions was determined by Mann-Whitney test. *p < 0.05; ***p < 0.0001; ns, not significant. See also Figure S6.

Titan cells can be induced *in vitro* by a variety of methods.^37,47,49,50^ Using two separate protocols, we observed significant differences in titanization between the parental and M1 strains (Figures 6A and S6A). Although the parental strain produced large numbers of titan cells, the M1 strain failed to produce any larger cells and was indistinguishable from the *cac1Δ* mutant strain. Addition of 20 mM cAMP to the media was sufficient to drive an increase in cell size in the M1 strain, but none crossed the 10 μm threshold for titan cell classification (Figures 6B and S6B). M1 cells were, however, significantly more responsive to exogenous cAMP treatment than the Ftc555-1 *cac1Δ* mutants, further demonstrating differences between the *cac1-evo* allele and complete loss-of-function alleles.

We next assessed the ability of our strains to produce smaller seed-cell populations using an *in vitro* protocol consisting of a series of media shifts, culminating in 24 h of growth in pigeon guano medium (PGM), a robust, environmentally relevant inducer of seed cells (STAR Methods).^48^ The Ftc555-1 parental strain exhibited a drastic downward shift in cell size upon transfer from capsule-inducing medium (CAP; STAR Methods) into PGM, a phenotype similar to the reference strain KN99α (Figure 6C).^48^ M1 cells exhibited a subtle but significant downward shift upon transfer from CAP to PGM more similar to the *cac1Δ* mutant, which maintained a consistent cell size across the tested conditions. These titan and seed-cell experiments demonstrate that the M1 strain, carrying just a single-nucleotide mutation in the *CAC1* gene, is severely impaired in its ability to modulate cell size in response to both host-like and environmentally relevant cues.

### The *cac1-evo* allele causes decreased mortality in murine infection

**Figure 7.**
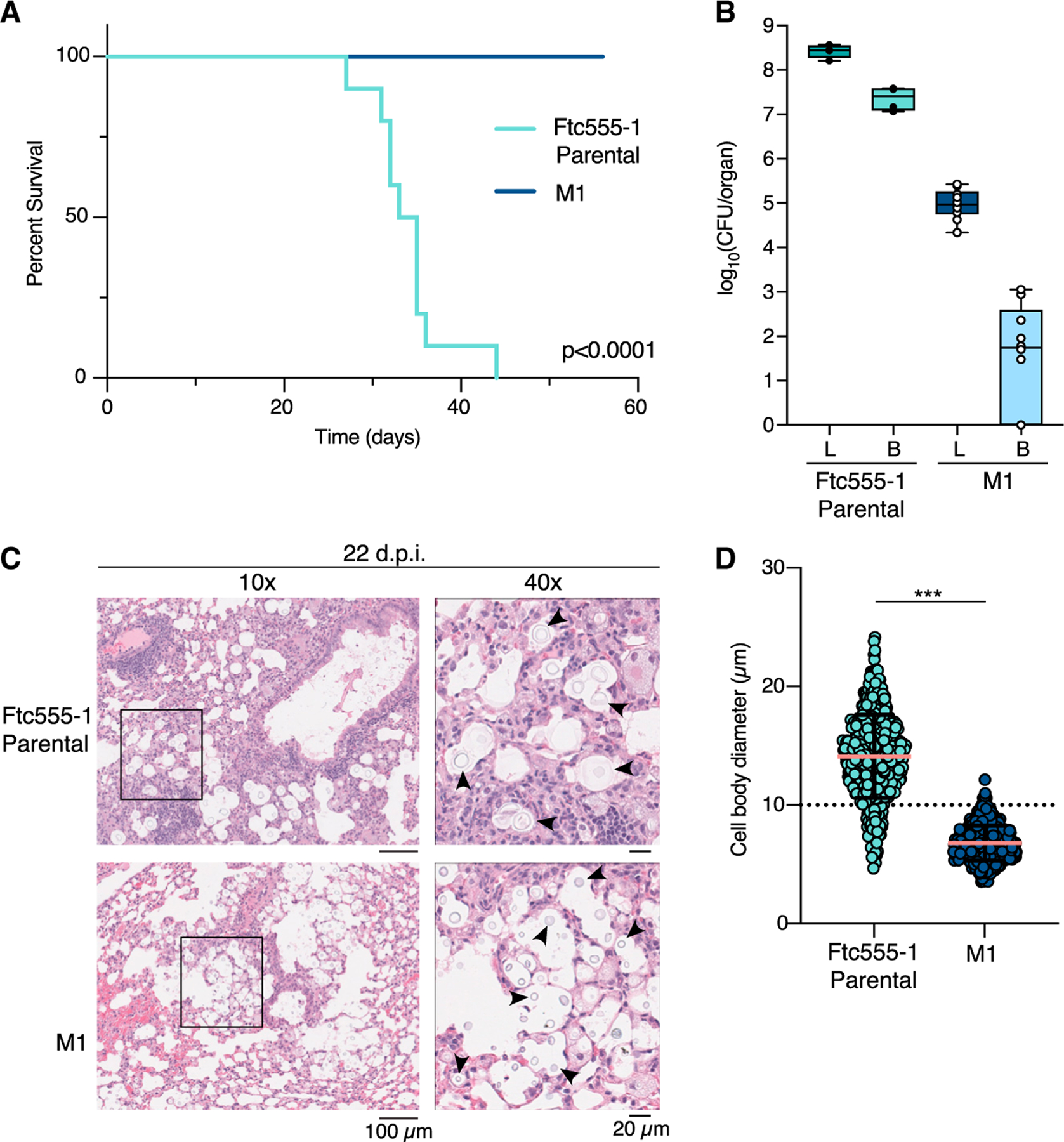
The cac1-evo allele decreases pathogenicity in murine infection models (A) Survival of C57BL/6NJ mice following intranasal inoculation with Ftc555-1 (teal) or M1 (navy blue). Ten animals were inoculated per strain (five male, five female) and were weighed daily until they reached predetermined endpoint criteria. Significance was determined by a log-rank test. (B) Fungal burden in the lungs (L) and brains (B) of infected mice from (A). Ftc555-1 parental values represent fungal burden at infection endpoint and M1 values are from organs harvested on day 56 post-infection. Each data point indicates the CFU count from an individual animal. Only a subset of the parental animals were analyzed for CFUs (four out of ten total infected mice). Boxes in box-and-whisker plots show the 25th to 75th percentiles. Whiskers show the full range of values (minimum to maximum) and the line indicates the median value. (C) Histopathological analysis reveals cell-size differences between animals infected with parental and M1 strains. Infected mouse lungs were harvested on day 22 post-infection, fixed, and stained with H&E. Positions of 40× images are indicated by the black box in 10× images. Black arrows indicate *C. neoformans* cells. Lungs from five animals per *C. neoformans* strain were analyzed and one representative image is shown. Scale bars, 100 μm (10× images) and 20 mm (40× images). (D) Quantification of *C. neoformans* cell size from H&E-stained lung sections. 100 cells per animal were analyzed and plotted. Line and error bars indicate mean ± SD. Significance was assessed by Mann-Whitney test. ***p < 0.0001. See also Figure S7.

Mutation of genes in the cAMP pathway, including *CAC1*, are associated with pathogenicity phenotypes *in vivo*.^33,35,36^ We used a murine model of disseminated cryptococcosis by inoculating C57BL/6NJ mice intranasally with either Ftc555-1 or the derived M1 strain to assess pathogenicity. The competitive advantages in intracellular macrophage replication we observe from *in vitro* assays with our evolved strain suggest a model where M1 might similarly replicate and disseminate to higher levels in the context of animal infection. In contrast, other phenotypic differences observed for the M1 strain predict a potential trade-off involving decreased fitness for this strain *in vivo* due to enhanced recognition and control by the immune system. Consistent with the second possibility, we observed a striking difference in survival between the animals infected with the two strains (Figure 7A). Animals infected with the parental strain, Ftc555-1, declined steadily after infection and had a median time to endpoint of 34 days, whereas the M1 strain failed to cause lethal infection or symptomatic disease in any animals during the experiment (Figures S7A and S7B).

To address whether the lack of disease in animals infected with M1 was due to clearance of *C. neoformans* after inoculation, we sacrificed animals 56 days post-infection and assessed fungal burden in both lung and brain tissue (Figure 7B). Intriguingly, we recovered CFUs from both the lungs and the brains of M1-infected mice; CFU levels from the lungs were, on average, higher than the starting inoculum (2.5 × 10^4^ fungi/mouse), although still much lower than in animals infected with the parental strain at infection endpoint. CFUs recovered from the brain were also low, although we observed dissemination to the brain in seven out of ten of the M1-infected animals. These results indicate that mice were persistently infected with the M1 strain, which maintains its potential for dissemination.

We subsequently performed histopathological analysis of the lungs from infected mice at 22 days post-infection—a time point prior to the first endpoints in animals infected with the parental strain (Figure 7C). Abundant cryptococcal cells were easily identified in the lungs, and there was not a pronounced difference in the distribution of these cells or the amount of tissue inflammation between mice infected with the parental or M1 strains. However, we observed significant cell-size variation between *C. neoformans* strains in these tissues (Figures 7C, 7D, and S7C). Consistent with *in vitro* observations, cells of the Ftc555-1 parental strain in fixed lung sections were nearly uniformly greater than 10 μm in all animals, whereas very few M1 cells crossed this threshold. Intriguingly, the smaller cell size of the M1 cells does not seem to be correlated, as predicted, with obvious decreases in cell number or with gross changes in host immune responses within the lungs. Furthermore, immune suppression of animals infected with the M1 strain by administration of the steroid dexamethasone daily for 14 days did not lead to any animals reaching endpoint, suggesting that fungal growth, rather than the host immune system, may be the limiting factor in M1 infection (Figure S7D).

These seemingly paradoxical results show the remarkable effect a single-nucleotide change in the genome of a pathogen can have on the disease-causing potential of the organism. This surprising trade-off in adaptation to macrophages leading to decreased pathogenicity in mouse infections raises new questions about the role different environments play in shaping the evolution of fungal pathogens and how this may impact the course and outcome of an infection.

## DISCUSSION

An emerging body of work examining strain-level variation both *in vitro* and *in vivo* reveals that even closely related *C. neoformans* strains can have divergent phenotypes.^4,5,7,9,10,47,51,52^ Our study provides a new perspective on phenotypic variation by directly comparing the growth of the same panel of clinicaland environmental isolates in host cells from two different species (Figures 1B–1E). We noted a correlation between strains that were able to withstand killing by amoebae and those that replicated well in macrophages, which suggests similarities in the intracellular environments of diverse host species that favor the growth of certain *C. neoformans* isolates over others. In contrast, experimental evolution revealed adaptive phenotypes that appear host specific, conferring an advantage in either amoebae hosts or in mammalian macrophages, but not both (Figure 2). Parallel studies of amoeba resistance, also in the Ftc555-1 background, similarly showed a lack of correlation between growth success in different host cells.^52,53^ Collectively, this suggests that although there may be some overlap in genetic pathways required for growth in evolutionarily diverse hosts, such overlap may be challenging to identify. The host-adapted isolates we recovered here along with future studies of non-laboratory strainswillhelpshed lighton conservation and divergence in host selective pressures that have shaped *C. neoformans* adaptive evolution.

Our courses of experimental evolution revealed that adaptation can happen over extremely short timescales in *C. neoformans* (Figure 2). Rapid adaptation of pathogens to new hosts or new environments is well documented for viral and bacterial pathogens.^54–57^ However, most previous studies of host adaptation in pathogenic fungi have focused on evolution occurring over much longer timescales.^25–27,58^ We estimate that the macrophage-passaged lines generated here were evolved for roughly 75–100 total generations, whereas amoeba-passaged *C. neoformans* strains experienced fewer generations due to potent killing by amoeba in early passages. Preliminary sequencing data revealed unique adaptive changes in our host-adapted populations, whereas other populations failed to adapt at all, highlighting the power of serial-passaging approaches to reveal a range of evolutionary trajectories.

The exact effect of the single point mutation we discovered in Cac1 and the mechanism by which this contributes to the growth phenotypes observed in M1 remains an exciting open question. Dissection of the functional impact of the *cac1-evo* allele in our adapted strain revealed unforeseen complexity in the regulation of cAMP signaling and downstream pathogenicity-related phenotypes. Our analysis of phenotypic impacts of the *cac1-evo* allele and the responsiveness of different strains to cAMP supplementation reveals two important findings: first, the Arg1227Pro mutation likely results in decreased cAMP production. Second, cAMP production differs across both the strains and experimental conditions tested, suggesting that even subtle changes in this signaling pathway may have profound effects on phenotypic outcome (Figures 5, 6, S5, and S6). How exactly environmental cues get integrated and through what machinery to affect cAMP and strain behavior *in vitro* and *in vivo* are important outstanding questions. Future genetic and biochemical analyses of *the cac1-evo* allele will explore the complex impact of this point mutation in *CAC1*.

It is also important to note that previous studies of cAMP signaling generally and *CAC1* specifically were carried out almost exclusively in the context of the laboratory strains H99 and KN99. Two notable exceptions are studies of *PKR1* and *PDE2* variation in clinical isolates of *C. neoformans*, with demonstrated impacts on titan-cell formation and virulence.^47,59^ Work here revealed both functional differences in the cAMP signaling pathway components and substantial sequence variation across *CAC1* sequences from diverse *C. neoformans* isolates, despite conservation at Arg1227 (Figure S5). In the Ftc555-1 parental strain alone, there are 17 coding changes in *CAC1* compared with the most studied H99 lineage strains. Gene trees of *CAC1* are incongruent with strain-level phylogenies, hinting at differences in the evolutionary pressures and histories of this gene across diverse strains (Figure S5A). Therefore, there may be functionally significant differences in the activity of this critical signaling pathway due to sequence changes in *CAC1* itself or through epistasis with other genes with altered sequences or activity among strains. Curiously, *PKR1* mutations have also been identified in serially sampled isolates from human patients, as well as in amoebae-adapted strains, although the impact of those mutations on adaptive phenotypes remains to be tested.^24,53^ Regardless, the convergence of multiple studies, including ours, on the cAMP signaling pathway hint that it is a hotspot of genetic variation within this species, with functional implications for a range of pathogenicity-associated phenotypes. This underscores the power and importance of using naturally occurring genetic variation among non-reference strains to gain insights into key virulence pathways that have previously only been studied in a single genetic context.^60^

Our results raise intriguing questions about the relative trade-offs between adaptation to specific host-cell environments and the ability to cause disease. Mouse infections revealed a striking difference in the pathogenicity of the serially passaged M1 strain when compared with the ancestral Ftc555-1 (Figure 7). Given its enhanced ability to replicate in mouse macrophages, we were surprised that M1 failed to cause lethal disease. However, the data are consistent with the important role of cell-size variation in modulating infection. Our analyses of cell-size changes in the M1 strain revealed that the *cac1-evo* allele functionally locks cells as a single morphotype (Figures 6, 7, S6, and S7). The inability of the M1 strain to generate cell-size heterogeneity partially explains the lack of severe disease in animals infected with this strain. However, further characterization of the M1 cells and their macrophage interactions may help inform our understanding of persistence and dissemination during animal infections, despite this lack of cell-size plasticity.

Finally, histopathologic analysis of the lungs from infected animals showed less pronounced differences than expected considering the distinct outcomes of infections caused by parental and evolved strains. Future work more closely examining host cytokine production, immune cell recruitment, and disease progression during infection with this strain will help shed light on interactions with the host immune system and how this contributes to persistent infection in the absence of obvious disease. Regardless of the exact mechanism, our work shows the complex impact of a single-nucleotide change in the genome of this pathogenic fungus and highlights the long reach of simple adaptive changes to fundamentally change the course of evolution.

## STAR★METHODS

### RESOURCE AVAILABILITY

#### Lead contact

Further information and requests for resources and reagents should be directed to and will be fulfilled by the lead contact, Nels Elde (nelde@genetics.utah.edu).

#### Materials availability

All strains and materials generated for this paper are available without restriction. Please request from the lead contact.

#### Data and code availability

- All DNA sequencing datasets have been deposited in the NCBI SRA and are publicly available. The BioProject accession number is listed in the key resources table. The paper also analyzes existing, publicly available data. These accession numbers are also listed in the key resources table. Raw histopathology data from this study are hosted on the HistoWiz website and are publicly available as of the date of this publication. Accession link is provided in the key resources table. All other data reported in this paper will be shared by the lead contact upon request.
- This paper does not report original code. Existing bioinformatic packages were used as specified in the method details and the key resources table lists version numbers.
- Any additional information required to reanalyze the data reported in this paper is available from the lead contact upon request.

### EXPERIMENTAL MODEL AND SUBJECT DETAILS

#### Fungal strains and growth media

*C. neoformans* strains used in this study are listed in the key resources table. Strains were stored at −80°C in yeast extract peptone dextrose (YPD) media supplemented with 20% glycerol. Strains were inoculated by streaking onto YPD plates and incubated for 2–3 days at 30°C before use in experiments. Strains were stored at 4°C for <10 days and reinoculated as needed. Unless otherwise noted, all overnight cultures were grown for 16–18 hours in YPD at 30°C with shaking at 225 rpm.

The following media types were used for *C. neoformans* growth in this study: YPD (Yeast Extract [Gibco Cat# 212750], Proteose Peptone [Gibco Cat# 211677], 2% glucose), YNB (yeast nitrogen base without amino acids [Sigma Cat# Y0626], 2% glucose), 10% Sab’s/CAP media (10% Sabouraud Dextrose Broth [Difco Cat# 238230], buffered with 50 mM HEPES to pH=7.4), tissue culture media (DMEM with high glucose and L-glutamine [VWR Cat# 16777-129] supplemented with 10% heat-inactivated FBS, non-essential amino acids and penicillin-streptomycin), RPMI 1640 (VWR Cat# 16777-145), and RPMI 1640 with L-glutamine and without sodium bicarbonate (Gibco Cat# 31800089).

#### Macrophage cell culture

J774A.1 cells (female) were purchased from ATCC (ATCC Cat# TIB-67, RRID: CVCL_0358). Cells were cultured in a humidified incubator at 37°C with 5% CO_2_ in DMEM with high glucose and L-glutamine (VWR Cat# 16777-129) supplemented with 10% heat inactivated FBS, non-essential amino acids and penicillin-streptomycin. J774A.1 macrophages were used between passages 5 and 20 from thaw for all experiments. Cells were routinely tested for mycoplasma contamination. Cell lines were not authenticated.

#### Amoeba cell culture

The *Acanthamoeba castellanii* amoeba strain ATCC 30234 was purchased from ATCC and cultured axenically in Peptone-Yeast-Glucose (PYG) Media (2% proteose peptone, 0.1% yeast extract, 0.1 M glucose, 4 mM MgSO_4_, 0.4 M CaCl_2_, 0.1% sodium citrate dihydrate, 0.05 mM Fe(NH_4_)_2_(SO_4_)_2_• 6H_2_O, 2.5 mM NaH_2_PO_3_, 2.5 mM K_2_HPO_3_, pH 6.5). Unless otherwise noted, all chemicals were purchased from Sigma. Amoebae were routinely cultured in T175 tissue culture flasks at 28°C and were used for assays between passages 5 and 20 from thaw.

#### Mouse infection models

For survival experiments, ~8-week-old C57BL/6NJ mice were ordered from Jackson Laboratory (RRID: IMSR_JAX:005304) and randomly assigned to experimental groups, with 5 male and 5 female mice tested per condition. Mice were anesthetized with ketamine/dexmedetomidine hydrochloride (Dexdomitor, Zoetis) delivered intraperitoneally. They were then suspended by their front incisors on a horizontal strand of thread. Mice were inoculated intranasally with 2.5x10^4^ *Cryptococcus* cells in 50 μl of PBS using a micropipette. The inoculum was placed dropwise onto a nasal flare before being inhaled by mice. Ten minutes later, mice were intraperitoneally administered the reversal agent atipamezole (Antisedan, Zoetis). Mice were weighed daily and euthanized by CO_2_ asphyxiation and cervical dislocation when they lost 15% of their initial mass.

For histopathology analysis, 5 ~8-week-old C57BL/6NJ female mice were infected as above. At 22 d.p.i., all animals were humanely euthanized.

For the immune suppression experiment, 10 ~8-week-old C57BL/6NJ female mice were infected with the M1 macrophage-adapted strain as above. Mice were monitored for 36 days and were then treated with either PBS (n=5) or 1mg/kg/day dexamethasone (n=5, Sigma Cat# D4902). PBS and dexamethasone were delivered intraperitoneally daily and animals were monitored for weight loss for 14 days and were then euthanized as above.

All animal procedures were approved by the University of Utah Institutional Animal Care and Use Committee.

### METHOD DETAILS

#### Co-culture of fungi and macrophage or amoeba

Macrophages were seeded into two replicate 96-well plates 18–24 hours before each assay at a density of 10^4^ cells/well in 100 μL normal macrophage growth media and incubated at 37°C and 5% CO_2_. Overnight cultures of each *C. neoformans* strains were subcultured down to an OD of 0.2 and allowed to reach mid-log phase (4–5 hours, OD=0.6–1.0) before use in experiments. Each strain to be tested was then washed twice with sterile PBS and adjusted to a concentration of ~2x10^5^ cells/mL in macrophage growth media containing 1 μg/mL of the anti-GXM antibody 18B7 (Sigma Cat# MABF2069). Cells were opsonized in this media for 1h at 37°C. While *C. neoformans* cells were opsonizing, macrophages were activated for 1 hour by replacing the media with 100 μL of growth media with 10 nM phorbol myristate acetate (PMA, Sigma Cat# P1585). Media was removed from activated macrophages and 100 μL of opsonized *C. neoformans* suspensions were added to each well (2x10^4^ yeast cells/well for an MOI ~1). 100 μL of opsonized cell suspensions were also added to empty wells in the plate as controls for media growth. *C. neoformans* cells were allowed to be phagocytosed for 1 hour with incubation at 37°C with 5% CO_2_. After 1 hour, supernatant and extracellular yeasts were removed and macrophages were gently washed 1–2 times with warm PBS before replacing with fresh macrophage growth media. One replicate plate was immediately lysed with 200 μL of 0.1% sodium deoxycholate to determine starting CFU and percent phagocytosis counts. Wells were washed twice with sterile water to collect all *C. neoformans* cells and combined with lysates. The remaining plate was incubated for an additional 24 hours before lysis. Lysed cultures were serially diluted and plated onto YPD. Plates were incubated at 30°C for 2–3 days before CFUs were enumerated.

Fold change in CFU was calculated by comparing the CFU counts after 1 hour of phagocytosis to the 24 hour CFU counts. Media only fold changes were calculated by comparing CFU counts from the media only wells at 1 hour vs. the media only wells at 24 hours. Percent phagocytosis was determined by comparing the 1 hour CFU counts from wells with macrophages to the control wells with media only.

Amoeba co-culture experiments were carried out similarly to the macrophage experiments with a few modifications. Amoeba were seeded at a density of 5x10^4^ cells/well into replicate 96-well plates and *C. neoformans* cell suspensions were prepared at 1x10^6^ cells/mL in Ac Buffer (4 mM MgSO_4_, 0.4 M CaCl_2_, 0.1% sodium citrate dihydrate, 0.05 mM Fe(NH_4_)_2_(SO_4_)_2_• 6H_2_O, 2.5 mM NaH_2_PO_3_, 2.5 mM K_2_HPO_3_, pH 6.5) to maintain an MOI of ~1. Amoeba were starved of nutrients in Ac Buffer for 1 hour at 28°C before 100 μL of yeast cell suspensions were added, and the cultures remained in Ac Buffer throughout the duration of the experiment. All incubations were performed at 28°C. Timings, washes, and CFU enumeration was carried out as described above. For all co-incubation experiments, each condition and strain was tested in triplicate and experiments were repeated 2–3 times on different days.

#### Serial passaging of fungi through host cells

8.5–9 x 10^5^ macrophage or *A. castellanii* cells were seeded into T25 flasks 16–18 hours before each passage and incubated under their respective normal growth conditions. For the first passage, an overnight culture of Ftc555-1 was grown with shaking for 16 hours with shaking at 30°C. This culture was centrifuged at 3000 x g for 5 minutes and washed twice with PBS before the OD was measured. Cells were normalized to an OD of 1 (~1.5 x 10^7^ cells/mL) in 3.5 mL of either macrophage growth media containing 1 μg/mL 18B7 anti-capsule antibody (for macrophage passaging) or in Ac Buffer (for amoeba passaging). Cell suspensions were incubated at 37°C (macrophage passaged) or 28°C (amoeba passaged) for 1 hour. The remaining starting inoculum culture was mixed 1:1 with 40% glycerol and aliquots were frozen at −80°C.

While *C. neoformans* cells were incubating, the media from macrophage containing flasks was removed and replaced with macrophage growth media containing 10 nM PMA. PYG media was removed from amoeba flasks and replaced with Ac buffer and cells were incubated at their standard temperatures for 1 hour. Following this 1 hour incubation, 1 mL of the appropriate *C. neoformans* cell suspension was added to each flask and gently rocked to ensure the entire host cell layer was covered. This small volume ensured that yeast cells settled onto the host cell monolayer and facilitated phagocytosis. Phagocytosis was allowed to proceed for 2 hours before extracellular yeasts were removed. Cells were washed 1–2 times with pre-warmed PBS and media was replaced with 2 mL of growth media. Phagocytosed yeasts and host cells were incubated for 24 hours.

After 24 hours of incubation, the media containing extracellular yeasts was discarded from lines 1 and 2 of both amoeba- and macrophage-containing flasks (A1-A2 and M1-M2). One mL of 0.1% sodium deoxycholate was added to all of the flasks to lyse host cells and lysates were collected. Flasks were washed twice with PBS. Lysates and washes were combined and centrifuged at 3000 x g for 5 min and the supernatant was discarded. *C. neoformans* cell pellets were resuspended in YPD media, vortexed to mix, and incubated at 30°C with shaking (225 rpm) for 24 hours.

Following outgrowth, ODs were measured and each individual culture was washed and adjusted to an OD of 0.425 in 1.5 mL of the appropriate growth media. The remaining culture was frozen as above to create a fossil record for the experiment. Passaging proceeded as above from this point. All macrophage passages were performed at 37°C with 5% CO_2_. Amoeba passages were largely carried out at 28°C, but passages 5 and 10 were performed at 37°C with 5% CO_2_ to avoid losing thermotolerance in these strains. All outgrowth steps were carried out at 30°C. Following the outgrowth of the final passage, multiple aliquots were frozen. Samples from each population were also serially diluted and plated onto YPD plates for CFU determination. Individual colonies from these plates were picked into 5 mL of YPD, grown overnight and frozen for use in phenotyping experiments.

#### Strain construction and molecular biology

Primers used in this work are listed in Table S1. All genetic manipulations were done using CRISPR-mediated gene editing and the TRACE system following previously published protocols.^63,79^ Briefly, gRNAs were generated using guide-specific primers and amplification from the pBHM2329 plasmid (gift from H. Madhani). *CAS9* was amplified for transformation from the pBHM2403 plasmid. All DNA used for CRISPR transformations was generated by PCR and purified using the Zymo DNA Clean and Concentrator 25 kit. If necessary, ethanol precipitations were performed to further concentrate DNA. Cells were grown as described for transformations and DNA was introduced by electroporation with an Eppendorf Eporator at 2 kV. DNA concentrations used were as follows, unless otherwise noted: 250 ng Cas9, 100 ng gRNA, 2.5 μg Repair Template.

For insertion of a the nourseothricin cassette at the Safe Haven 2 locus (SH2), we designed a VNB lineage-specific guide targeting this locus.^80^ A repair template plasmid carrying the ~1 kb of sequence immediately 5’ and 3’ to this gRNA sequence from the H99 genome was constructed using HiFi DNA Master Mix (NEB Cat#E2621) with a standard NAT^R^ cassette inserted between the two flanks. Repair templates were then PCR amplified from this plasmid (pZAH31) for CRISPR transformations.

For allele swap strains, the M1-derived mutation in *CAC1* conveniently generates a new PAM site in this strain, so we used strain-specific gRNAs that overlap or terminate at this mutation, wherein the PAM and guide sequences would then be disrupted by introduction of the alternate allele. Repair constructs containing the site of the mutation and ~900 bp upstream and downstream of this position were amplified directly from genomic DNA from either Ftc555-1 or M1. We used a co-CRISPR approach, simultaneously targeting *CAC1* and the SH2 locus in order to be able to select for successful transformation. For allele swap transformations, we simultaneously transformed the following amounts of DNA: 250 ng M1- or Ftc555-1-specific gRNA, 250 ng VNB SH2 gRNA, 1 mg Cas9, 2 mg *CAC1* gDNA Repair Template, 2 mg SH2-NAT Repair Template.

For generating cAMP pathway gene mutants in the Ftc555-1 and M1 backgrounds, gRNAs targeting the 5’ and 3’ ends of the gene were generated and both were used in transformations to ensure deletion of the entire open-reading frame. A repair template plasmid was created by amplifying the ~1kb regions upstream and downstream of the ORF of *CAC1, ACA1, GPA1 or GPR4* from Ftc555-1 gDNA. HiFi DNA master mix was used to insert a hygromycin or nourseothricin resistance cassette (HYG^R^ or NAT^R^) between these flanking gDNA sequences and ligate it into pUC19. Repair template was amplified from the resulting plasmid for use in CRISPR transformations. Plasmids generated are listed in the key resources table.

#### Transformant selection and genotyping

Following electroporation of CRISPR reagents, strains were plated onto selective media (either YPAD+NAT or YPAD+HYG) and allowed to grow for 2–3 days at 30°C. Individual colonies were then patched onto non-selective YPD media for 2–3 passages before being patched back onto selective media in order to identify stable genomic integrants.

Colonies were genotyped by colony PCR: small amounts of cells were picked into PCR strip tubes and microwaved for 2.5 minutes before a Phusion-Flash (Thermo Cat# F548S) PCR mix containing the appropriate primers was added directly to these cells. For SH2 and gene knockout genotyping, a primer sitting inside of the inserted drug cassette on both 5’ and 3’ ends (ZAH_G40 and G01) were paired with primers in the flanking genomic regions outside of the repair template constructs (see Table S1). Allele swap colonies were genotyped using primers 5’ and 3’ to the repair construct (ZAH_H20 and H21) paired with destabilized primers designed with the WebSNAPER tool to bind specifically to either the parental or evolved *CAC1* allele (ZAH_H22 for the parental allele and ZAH_H30 for the evolved allele).^78^ Allele swap colonies were first genotyped for the correct *CAC1* allele, and were then confirmed to also carry the NAT^R^ cassette at the SH2 locus.

Genomic sequences were amplified from gDNA extracted from candidate colonies and integration and allele identity was confirmed by Sanger sequencing. PCRs were also performed to ensure that no strains had integrated the gRNA or *CAS9* constructs.

#### Competition experiments

For competition experiments, cell suspensions were made identically for test strains and the NAT^R^-labeled parental Ftc555-1. These cell suspensions were mixed 1:1 before adding 100 μL to wells for co-culture experiments, which were carried out as described above. To determine the CFUs for test strain vs. the parental strain, serial dilutions were plated onto both YPD plates as well as YPAD+NAT plates. Competitive index here is calculated as:

$$Competitive\;Index=\frac{\frac{24\;hr\;CFU_{Test\;Strain}}{24\;hr\;CFU_{\text{NAT}\;Control}}}{\frac{1\;hr\;CFU_{Test\;Strain}}{1\;hr\;CFU_{\text{NAT}\;Control}}}$$

#### Intra- and extracellular CFU determinations

To determine intracellular and extracellular CFU counts, co-incubation experiments were set up as described above. After 24 hours of incubation with macrophages, the media supernatant containing extracellular yeasts was removed to an empty well of the 96-well plate and replaced with fresh macrophage media. If there were still substantial unassociated yeasts observed, this step was repeated for a second time. Otherwise, macrophages were lysed as above to collect intracellular yeasts. Both intracellular and extracellular wells were washed, serially diluted, and plated for CFU enumeration as described.

#### Fluconazole MICs and competition

Overnight cultures of *C. neoformans* strains were adjusted to a density of ~5x 10^6^ cells/mL and were spread onto either YPD-agar or RPMI agar medium with L-glutamine and without sodium bicarbonate. Fluconazole Etest strips (bioMérieux) were placed on the agar surface of the RPMI plates once cultures had dried. All plates were placed at 37°C and imaged after 48 and 72 hours. Only strains with robust growth on control plates (-Etest strip) were imaged and used to determine MIC values.

For macrophage experiments and competitions with fluconazole supplementation, co-incubation setup was performed as above through the one hour phagocytosis step. When extracellular yeasts and media were removed, media was replaced with standard macrophage growth media containing either 2 μg/mL Fluconazole (in DMSO, Sigma Cat#F8929) or media with an equal volume of DMSO. The remainder of the experiment was performed as described above.

#### Genomic DNA extraction and sequencing

Single colonies or 250 μL of pooled glycerol stocks for sequencing were inoculated into 50 mL of YPD and grown overnight with shaking at 30°C. Cells were collected by centrifugation and lyophilized overnight. High-molecular-weight genomic DNA was isolated following the CTAB extraction protocol as previously described. Following CTAB extraction, samples were further purified using the Takara ChromaSpin 1000 column. Concentrations were measured by Qubit broad range assay. For Nanopore sequencing of Ftc555-1, one microgram of HMW gDNA was prepared for sequencing using the SQK-LSK110 Kit and run on an R9 (FLO-MIN106) flow cell. For Illumina sequencing, samples were sheared with a Covaris S2 Focused-ultrasonicator and libraries were prepared using the New England Biolabs NEBNext Ultra II DNA Library Prep kit (Cat#E7634L) with an average insert size of 450 bp. 12–14 strains per run were barcoded and pooled, and 100 million 150x150 bp paired-end reads were collected on a NovaSeq S4 Flow Cell with the v1.5 Reagent Kit. Illumina library preparation and sequencing was performed by the University of Utah’s High Throughput Genomics core facility at the Huntsman Cancer Institute.

#### Sequencing analysis

Fast5 files from Nanopore sequencing of the parental strain were re-basecalled using guppy-gpu (v 6.0.1). The resulting fastq files were directly used for assembly using Canu (v 2.1.1) with an estimated genome size of 20 Megabases and default parameters.^64^ This assembly had 14 chromosome-scale contigs and a number of shorter contigs which mapped either to telomeric regions or mitochondrial DNA. These were manually trimmed from the assembly. The assembly was polished once with Nanopore reads using Medaka (v 1.5.0; https://github.com/nanoporetech/medaka) and then polished five times with Illumina reads from the parental strain using pilon (v 1.24) until no additional changes were being made to the assembly.^65^ A circular mitochondrial genome was assembled from Illumina reads using GetOrganelle (v1.7.5.3) with the fungus_mt setting and confirmed by comparison to the trimmed contigs assembled by Canu.^66^ This mitochondrial genome was manually added to the assembly. Chromosomes were named based on comparison to other reference assemblies and annotations were transferred using Liftoff (v1.6.3).^67^

For Illumina sequencing data of evolved colonies and passages, adapters were trimmed using Trimmomatic (v 0.39) and reads were aligned to our *de novo* assembly using bwa-mem2 (v 2.2.1).^68,69^ Duplicates were marked and read groups added with Picard and the resulting files were sorted and indexed with SAMtools (v1.15.1).^71^ Variants were called using sorted BAM files with the Genome Analysis Tool Kit (HaplotypeCaller and GenotypeGVCF, v3.8) with the ploidy set to 1.^70^ Variants were filtered out based on the following criteria using GATK VariantFiltration: QD<2.0, FS > 60, MQ <40, GQ <50, DP <10. To remove called SNPs in homopolymeric runs likely caused by sequencing errors, we used the BCFtools isec function to identify unique SNPs in each sample when compared to those found in the parental strain sequencing.^71^ The predicted impacts of called variants were assessed using SnpEff (v 4.3t) using our *de novo* assembly as reference^72^.

To analyze allele frequencies of the *cac1-evo* allele, sorted BAM files from each passage were visualized in IGV and the number of reads containing the Arg1227Pro (Chromosome 8 of our *de novo* assembly: 1057343 G>C, or Chromosome 8 of the H99 reference assembly: 333567 C>G ) were counted and normalized to the total sequencing coverage at that position.^73^ All sequencing analysis was performed using resources through the University of Utah Center for High Performance Computing. All of the raw sequence data has been deposited under NCBI BioProject:PRJNA941895.

#### Structure prediction and conservation analysis

The structure of the Ftc555-1 Cac1 protein was modeled using AlphaFold (v2.1.2).^74^ The predicted structure with the highest confidence (ranked_0.pdb) is shown. The catalytic domain was annotated based on sequence homology to the *S. cerevisiae* adenylyl cyclase homolog, Cyr1. The first 870 amino acids of Cac1 were unstructured in the model and were manually trimmed for display in Figure 5A.

Fungal adenylyl cyclase orthologs were downloaded from FungiDB or NCBI Genbank and aligned using MAFFT.^81–84^ Nucleotide sequences for the *CAC1* genes from all publicly available *C. neoformans* strains were similarly downloaded from FungiDB. Introns were trimmed and the coding sequences were aligned in Geneious using the Translation Align function with the MUSCLE algorithm option. Alignments of the *CAC1* sequences from the strains used in this paper were used as input to IQ-TREE for phylogenetic tree construction.^75^ The MGK+F3X3+I model was selected by the ModelFinder function, and the tree was estimated with 100 non-parametric bootstrap replicates.^85^ Some IQ-TREE analyses were performed with the IQ-Tree webserver.^86^ Bootstrap values for the tree shown in Figure S5A were uniformly greater than 95 indicating high confidence in the depicted tree.

#### Melanization assays

Melanization of different strains was analyzed following previously established protocols.^87^ Briefly, overnight cultures of *C. neoformans* strains were pelleted by centrifugation (3000xg, 5 minutes), washed twice in sterile water and adjusted to an OD of 0.25. Five microliters of each strain was spotted onto L-DOPA plates (7.6 mM L-asparagine monohydrate, 5.6 mM glucose, 10 mM MgSO_4_, 0.5 mM 3,4-dihydroxy-L-phenylalanine, 0.3 mM thiamine-hydrochloride, and 20 nM biotin) and onto YPD plates and incubated at 30°C or 37°C. Plates were checked every day until the WT control (KN99α) turned dark brown, usually day 3. For cAMP supplementation experiments, cAMP (Cayman Chemical) was added to plates to a final concentration of 2.5 mM. Strain preparations were spotted in parallel onto cAMP containing and control L-DOPA plates.

#### *In vitro* capsule induction assays

Capsule was induced using two different protocols.^43^ For both protocols, *C. neoformans* strains of interest were grown overnight (16–18 hours) in YPD media and OD_600_ was measured for each strain. In the first protocol, strains were subcultured to an OD of 0.3 in DMEM media containing 10% FBS, non-essential amino acids, and penicillin/streptomycin and incubated in a 6-well plate at 37°C with 5% CO_2_. In parallel, the same subcultures were subcultured to an OD of 0.3 in 10% Sabouraud’s Dextrose Media with 50 mM HEPES adjusted to pH 7.4. Strains in 10% Sab’s were incubated in 6 well plates at 37°C without CO_2_. As a control, the same strains were diluted in YPD media to the same OD and replicate plates were incubated at 37°C with or without CO_2_. Cell and capsule size measurements were made after 48 hours of incubation. Cells were centrifuged at 3000 x g, resuspended in PBS and fixed with formaldehyde. Samples were then mixed 1:1 with india ink to visualize capsule. 5–10 μL was pipetted onto a microscope slide and pictures were taken on an AxioObserver 7 (Zeiss) at 100x magnification. For each strain, measurements were made for 50 cells under each condition using a combination of manual measurement in FIJI and the automated QCA Matlab program.^76,77^ For cAMP supplementation experiments, cAMP was dissolved in the same media used above at a concentration of 20 mM and the final pH of the media with and without cAMP was equalized.

#### Titan and seed cell induction assays

Titan cells were induced using two different previously described protocols: by incubation in serum-free RPMI or in PBS with 10% heat-inactivated fetal bovine serum (HI-FBS).^37,50^ Briefly, overnight cultures were grown with shaking for 16–18 hours in YPD. Cells were washed three times in PBS and adjusted to a concentration of 5x10^3^ cells/ml in each media type. 1 mL aliquots were dispensed into three replicate wells of a 24-well plate and incubated at 37°C with 5% CO_2_ for 7 days. Exogenous cAMP supplementation experiments were carried out as above.

Seed cell formation was assessed *in vitro* using previously described protocols.^48^ Strains of interest were grown in YNB media overnight with rotation at 37°C. YNB cultures were then diluted 1:50 in 10% Sabouraud’s Dextrose Media with 50 mM HEPES (pH 7.4, also referred to CAP media) and grown with rotation at 37°C for 24 hours. To induce seed cells, 2.5 mL of the Sab’s culture was subcultured into Pigeon Guano Media (PGM) and grown for 24h at 37°C with rotation. At every subculture step, aliquots of cells were fixed for cell size quantification as described above. For seed cell and titan formation assays, cell visualization, imaging and cell size quantifications were carried out as in the capsule induction experiments above.

#### Animal experiments

Animal infections were performed as described above (see mouse infection models). For fungal burden determination, organs were harvested either at infection endpoint (parental-infected animals) or at 56 days post infection (M1 infected animals), placed on ice, and homogenized in 5 ml of PBS. Serial dilutions of homogenates were plated on Sabouraud’s dextrose agar with 10 mg/mL gentamicin and 100 mg/mL carbenicillin. Plates were incubated at 30°C for 2–3 days before CFUs were counted to determine fungal burden per organ.

For histopathology analysis, all animals were humanely euthanized at 22 d.p.i. and their lungs were harvested and fixed in neutral buffered formalin for 72 hours before being transferred to 70% ethanol. Histology was performed by HistoWiz Inc. Samples were processed, paraffin-embedded, sectioned and 4 sections per animal were stained with hematoxylin and eosin (H&E). Whole slide scanning (40x) was performed on an Aperio AT2 (Leica Biosystems). Images from all processed slides can be accessed at the link in the key resources table. One slide per animal was analyzed and scored by a board-certified veterinary pathologist through HistoWiz Inc.

### QUANTIFICATION AND STATISTICAL ANALYSIS

All statistical analyses were performed with GraphPad Prism 9 software. Information about quantification, replication and statistical details of experiments can be found in the corresponding figure legends.

## Supplementary Material

MMC1

## Figures and Tables

**Table:** KEY RESOURCES TABLE

REAGENT or RESOURCE	SOURCE	IDENTIFIER
Antibodies		

Mouse Anti-Glucuronoxylomannan (GXM) Antibody, clone 18B7	Sigma	Cat#MABF2069

Chemicals, peptides, and recombinant proteins		

cAMP	Cayman Chemical	Cat#18820
Phorbol myristate acetate (PMA)	Sigma	Cat#P1585
Phusion Flash DNA Polymerase	Thermo Fisher	Cat# F548S
HiFi DNA Master Mix	New England Biolabs	Cat#E2621
Fluconazole	Sigma	Cat#F8929
Hexadecyltrimethylammonium bromide (CTAB)	Sigma	Cat#H9151
3,4-dihydroxy-L-phenylalanine	Sigma	Cat#D9628
L-Asparagine monohydrate	Sigma	Cat#A7094
Thiamine hydrochloride	Sigma	Cat#T4625
Biotin	Sigma	Cat#B4639
Yeast Extract	Gibco	Cat#212750
Peptone	Gibco	Cat#211677
Yeast nitrogen base without amino acids	Sigma	Cat#Y0626
Sabouraud Dextrose Broth	Difco	Cat#238230
DMEM with high glucose and L-glutamine	VWR	Cat#16777-129
Fetal Bovine Serum	Thermo Fisher	Cat#26140079
RPMI 1640	VWR	Cat#16777-145
RPMI 1640 with L-glutamine w/o sodium bicarbonate	Gibco	Cat#31800089
Dexdomitor	Zoetis	Cat#122692-5
Antisedan	Zoetis	Cat#87219-02296-2
Dexamethasone	Sigma	Cat#D4902

Critical commercial assays		

NEBNext Ultra II DNA Library Prep	New England Biolabs	Cat#E7634L
Ligation Sequencing Kit	Oxford Nanopore	Cat#SQK-LSK110
Fluconazole Etest Strips	BioMerieux	Cat#412349
ChromaSpin 1000	Takara	Cat#636093

Deposited data		

Nanopore and Illumina sequencing data	Sequence Read Archive	NCBI BioProject: PRJNA941895
Histopathology images from mice infected with Ftc555-1 Parental and M1 strains	HistoWiz	HistoWiz Data:https://app.histowiz.com/shared_orders/897b4aa6-bf6c-49da-9009-7113f766aee9/slides/
*M. sympodalis* adenylyl cyclase sequence	NCBI Genbank	GenBank: SHO76694.1
*U. maydis* adenylyl cyclase sequence	NCBI Genbank	GenBank: XP_011388269
*C. neoformans* adenylyl cylase sequence	NCBI Genbank	GenBank: XP_012050872
*C. gattii* adenylyl cyclase sequence	NCBI Genbank	GenBank: XP_003195134.1
*S. pombe* adenylyl cyclase sequence	NCBI Genbank	GenBank: NP_596159.1
*N. crassa* adenylyl cyclase sequence	NCBI Genbank	GenBank: BAA00755.1
*B. cinerea* adenylyl cyclase sequence	NCBI Genbank	GenBank: XP_024553314.1
*T. rubrum* adenylyl cyclase sequence	NCBI Genbank	GenBank: KDB32528.1
*A. niger* adenylyl cyclase sequence	NCBI Genbank	GenBank:GAq42063.1
*C. albicans* adenylyl cyclase sequence	NCBI Genbank	GenBank:XP_716974.2
*S. cerevisiae* adenylyl cyclase sequence	NCBI Genbank	GenBank:NP_012529
*CAC1* sequences from all *C. neoformans* isolates	FungiDB	FungiDB: https://fungidb.org/fungidb/app/record/gene/CNAG_03202#category:dna-polymorphism

Experimental models: Cell lines		

J774A.1	ATCC	Cat# TIB-67; RRID: CVCL_0358

Experimental models: Organisms/strains		

Mice: C57BL/6NJ	Jackson Laboratory	RRID: IMSR_JAX:005304
Amoeba: *Acanthamoeba castellanii*	ATCC	Cat# 30234
*C. neoformans*: H99O	Gift from J. Perfect	N/A
*C. neoformans*: KN99α	Fungal Genetics Stock Center	Cat#10369
*C. neoformans*: KN99α *cac1Δ::NAT*	Fungal Genetics Stock Center, Madhani Knockout Collection (2020 Plates)	N/A
*C. neoformans*: Bt46	Gift from J. Perfect, Litvintseva et al.^61^	N/A
*C. neoformans*: Ze90-1	Gift from J. Perfect, Litvintseva et al.^62^	N/A
*C. neoformans*: Ftc217-1	Gift from J. Perfect, Chen et al.^5^	N/A
*C. neoformans*: Ftc239-1	Gift from J. Perfect, Chen et al.^5^	N/A
*C. neoformans*: Ftc209-1	Gift from J. Perfect, Chen et al.^5^	N/A
*C. neoformans*: Ftc209-3	Gift from J. Perfect, Chen et al.^5^	N/A
*C. neoformans*: NRHc5004.ENR	Gift from J. Perfect, Chen et al.^5^	N/A
*C. neoformans*: Muc458-2	Gift from J. Perfect, Chen et al.^5^	N/A
*C. neoformans*: NRHc5026.ENR.CLIN.ISO	Gift from J. Perfect, Chen et al.^5^	N/A
*C. neoformans*: Ftc555-1	Gift from J. Perfect, Chen et al.^5^	N/A
*C. neoformans*: Ftc158-2	Gift from J. Perfect, Chen et al.^5^	N/A
*C. neoformans*: NRHc5029.ENR.CLIN.ISO	Gift from J. Perfect, Chen et al.^5^	N/A
*C. neoformans*: Gbc39-1	Gift from J. Perfect, Chen et al.^5^	N/A
*C. neoformans*: Ftc555-1 M1-1	This study	N/A
*C. neoformans*: Ftc555-1 M2-1	This study	N/A
*C. neoformans*: Ftc555-1 M3-2	This study	N/A
*C. neoformans*: Ftc555-1 A1-1	This study	N/A
*C. neoformans*: Ftc555-1 A2-1	This study	N/A
*C. neoformans*: Ftc555-1 A3-1	This study	N/A
*C. neoformans*: Ftc555-1 *SH2-NAT*	This study	ZAH_CN01
*C. neoformans*: Ftc555-1 Parental *cac1-evo(R1227P); SH2-NAT* isolate H1	This study	ZAH_CN03
*C. neoformans*: Ftc555-1 Parental *cac1-evo(R1227P); SH2-NAT* isolate H7	This study	ZAH_CN04
*C. neoformans*: Ftc555-1 M1 *CAC1-parent (P1227R); SH2-NAT* isolate M2	This study	ZAH_CN05
*C. neoformans*: Ftc555-1 M1 *CAC1-parent (P1227R); SH2-NAT* isolate H11	This study	ZAH_CN06
*C. neoformans*: Ftc555-1 Parental *cac1Δ::HYG*	This study	ZAH_CN07
*C. neoformans*: Ftc555-1 M1 *cac1Δ::HYG; cac1-evo(R1227P)*	This study	ZAH_CN08
*C. neoformans*: Ftc555-1 Parental *aca1Δ::NAT*	This study	ZAH_CN09
*C. neoformans*: Ftc555-1 Parental *gpa1Δ::NAT*	This study	ZAH_CN10
*C. neoformans*: Ftc555-1 Parental *gpr4Δ::NAT*	This study	ZAH_CN11
*C. neoformans*: Ftc555-1 M1 *aca1Δ::NAT; cac1-evo(R1227P)*	This study	ZAH_CN12
*C. neoformans*: Ftc555-1 M1 *gpa1Δ::NAT; cac1-evo(R1227P)*	This study	ZAH_CN13
*C. neoformans*: Ftc555-1 M1 *gpr4Δ::NAT; cac1-evo(R1227P)*	This study	ZAH_CN14

Oligonucleotides		

See Table S1	N/A	N/A

Recombinant DNA		

*SH2::NAT* plasmid for CRISPR repair amplification	This study	pZAH31
*cac1Δ::HYG* plasmid for CRISPR KO repair template	This study	pZAH40
*aca1Δ::NAT* plasmid for CRISPR KO repair template	This study	pMWS05
*gpa1Δ::NAT* plasmid for CRISPR KO repair template	This study	pMWS06
*gpr4Δ::NAT* plasmid for CRISPR KO repair template	This study	pMWS07
*pRS316-PCnU6-scaffold* for sgRNA Fusion PCRs	Huang et al.^63^	AddGene Cat#171686; pBHM2329
*pRS316-PTEF1-CnoCas9* Codon Optimized *C. neoformans* Cas9 for CRISPR	Huang et al.^63^	AddGene Cat#171687; pBHM2403

Software and algorithms		

Guppy-gpu basecaller (v 6.0.1)	Oxford Nanopore	RRID: SCR_023196
Canu (v 2.1.1)	Koren et al.^64^	RRID: SCR_015880; https://github.com/marbl/canu
Medaka (v 1.5.0)	Oxford Nanopore	https://github.com/nanoporetech/medaka
Pilon (v 1.24)	Walker et al.^65^	RRID: SCR_014731; https://github.com/broadinstitute/pilon/
GetOrganelle (v 1.7.5.3)	Jin et al.^66^	https://github.com/Kinggerm/GetOrganelle
Liftoff (v 1.6.3)	Shumate and Salzberg^67^	https://github.com/agshumate/Liftoff
Trimmomatic (v 0.39)	Bolger et al.^68^	RRID: SCR_011848; http://www.usadellab.org/cms/index.php?page=trimmomatic
bwa-mem2 (v 2.2.1)	Vasimuddin et al.^69^	RRID: SCR_022192; https://github.com/bwa-mem2/bwa-mem2
Picard	Broad Institute	RRID: SCR_006525; https://github.com/broadinstitute/picard
Genome Analysis Tool Kit (GATK, v 3.8)	McKenna et al.^70^	RRID: SCR_001876; https://software.broadinstitute.org/gatk/
SAMtools (v 1.15.1)	Danecek et al.^71^	RRID: SCR_002105; https://www.htslib.org/
SnpEff (v 4.3t)	Cingolani et al.^72^	RRID: SCR_005191; https://pcingola.github.io/SnpEff/
Integrated Genomics Viewer (IGV)	Thorvaldsdóttir et al.^73^	RRID: SCR_011793; http://www.broadinstitute.org/igv/
AlphaFold (v 2.1.2)	Jumper et al.^74^	https://github.com/deepmind/alphafold
Geneious	Geneious	RRID: SCR_010519; http://www.geneious.com/
IQ-Tree	Nguyen et al.^75^	RRID: SCR_017254; http://www.iqtree.org/
FIJI	Schindelin et al.^76^	RRID: SCR_002285; https://fiji.sc/
QCA-Quigly’s Circle App	Dragotakes and Casadevall^77^	https://github.com/quigly555/QCA
Prism 9	GraphPad	RRID: SCR_002798; https://www.graphpad.com/
WebSNAPER	Drenkard et al.^78^	https://pga.mgh.harvard.edu/cgi-bin/snap3/websnaper3.cgi

## Highlights

- *C. neoformans* strains vary in their interactions with diverse host species
- Serial passaging reveals that fungi adapt rapidly to distinct host-cell environments
- A missense mutation in adenylyl cyclase confers increased growth in macrophages
- Adaptation to macrophages comes with trade-offs for *in vivo* pathogenicity
