# Supplementary material for: Distinct pathways of adaptive evolution in Cryptococcus neoformans reveal a mutation in adenylyl cyclase with trade-offs for pathogenicity
Source: Curr Biol. Author manuscript; Available in PMC 2023 Oct 23. (PMC10592076; doi:10.1016/j.cub.2023.08.054)
Supplement: MMC1 [file NIHMS1928360-supplement-MMC1.pdf]

Current Biology, Volume *33*

Supplemental Information

Distinct pathways of adaptive evolution in *Cryptococcus neoformans* reveal a mutation in adenylyl cyclase with trade-offs for pathogenicity

Zoë A. Hilbert, Joseph M. Bednarek, Mara J.W. Schwiesow, Krystal Y. Chung, Christian T. Moreau, Jessica C.S. Brown, and Nels C. Elde

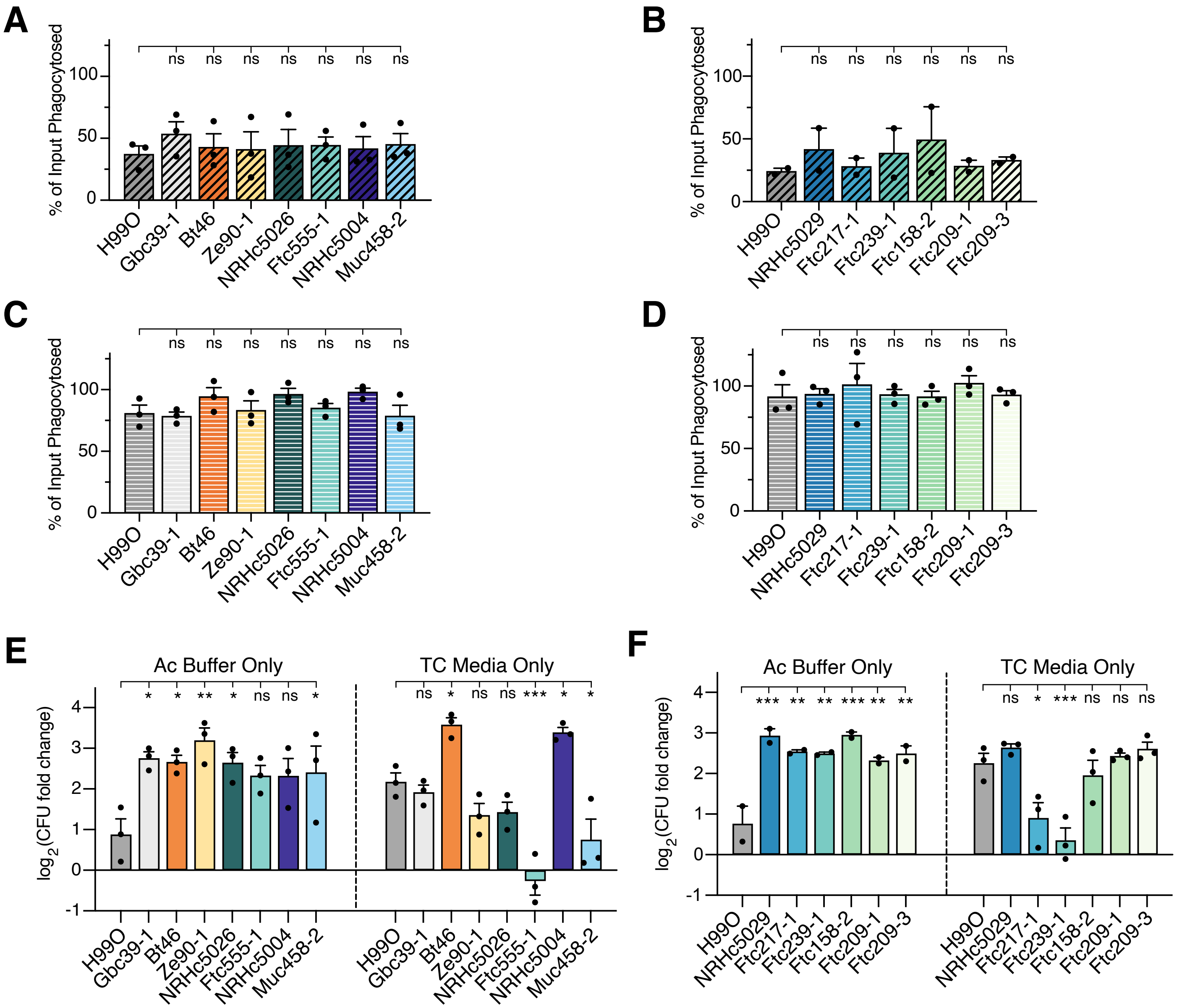

Figure S1. ***C. neoformans*** **clinical and environmental isolates vary in media growth phenotypes but not in phagocytosis rates in amoebae and macrophages. Related to Figure 1.** The percent of cells from the input culture that were phagocytosed over one hour of incubation with *A. castellanii* cells (A and B) or mouse macrophages (C and D) was calculated as the number of CFUs recovered after 1 hour of co-incubation with host cells divided by the number of CFUs from control media only wells (E and F) Strain growth over 24 hours in amoebae culture media (Ac Buffer, left) or tissue culture media (right) in the absence of host cells. Plotted data indicate the average values ± SEM from 2-3 independent experiments on different days. Each dot indicates the average of three replicate measurements from a single experiment. Significance in all panels was assessed by comparison to the laboratory strain H99O using ordinary one-way ANOVA followed by Dunnett's multiple comparisons test. *p<0.05, **p<0.001, ***p<0.0001, ns, not significant.

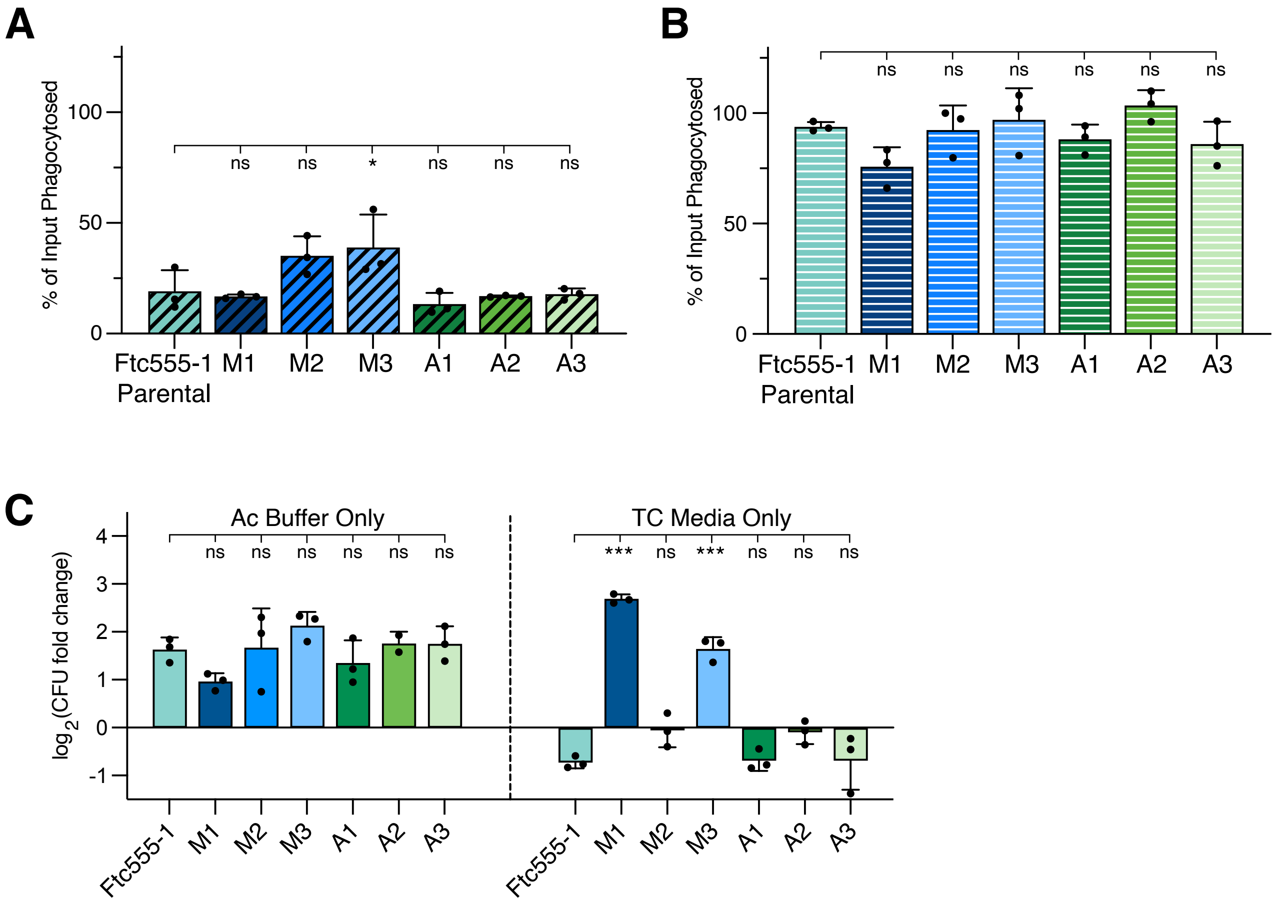

Figure S2. Evolved strains of Ftc555-1 have varied media growth differences and little alteration to phagocytosis rates. Related to Figure 2. The percent of cells from the input culture that were phagocytosed over one hour of incubation of *A. castellanii* cells (A) or mouse macrophages (B) calculated as in Figure S1. (C) Media only growth of evolved strains in Ac Buffer (left) or tissue culture media (right) in the absence of host cells. Plotted data indicate the average values ± SD from one representative experiment. Each dot represents one replicate from that experiment. Significance was assessed by comparison to the parental strain using ordinary one-way ANOVA followed by Dunnett's multiple comparisons test. *p<0.05, ***p<0.0001, ns, not significant.

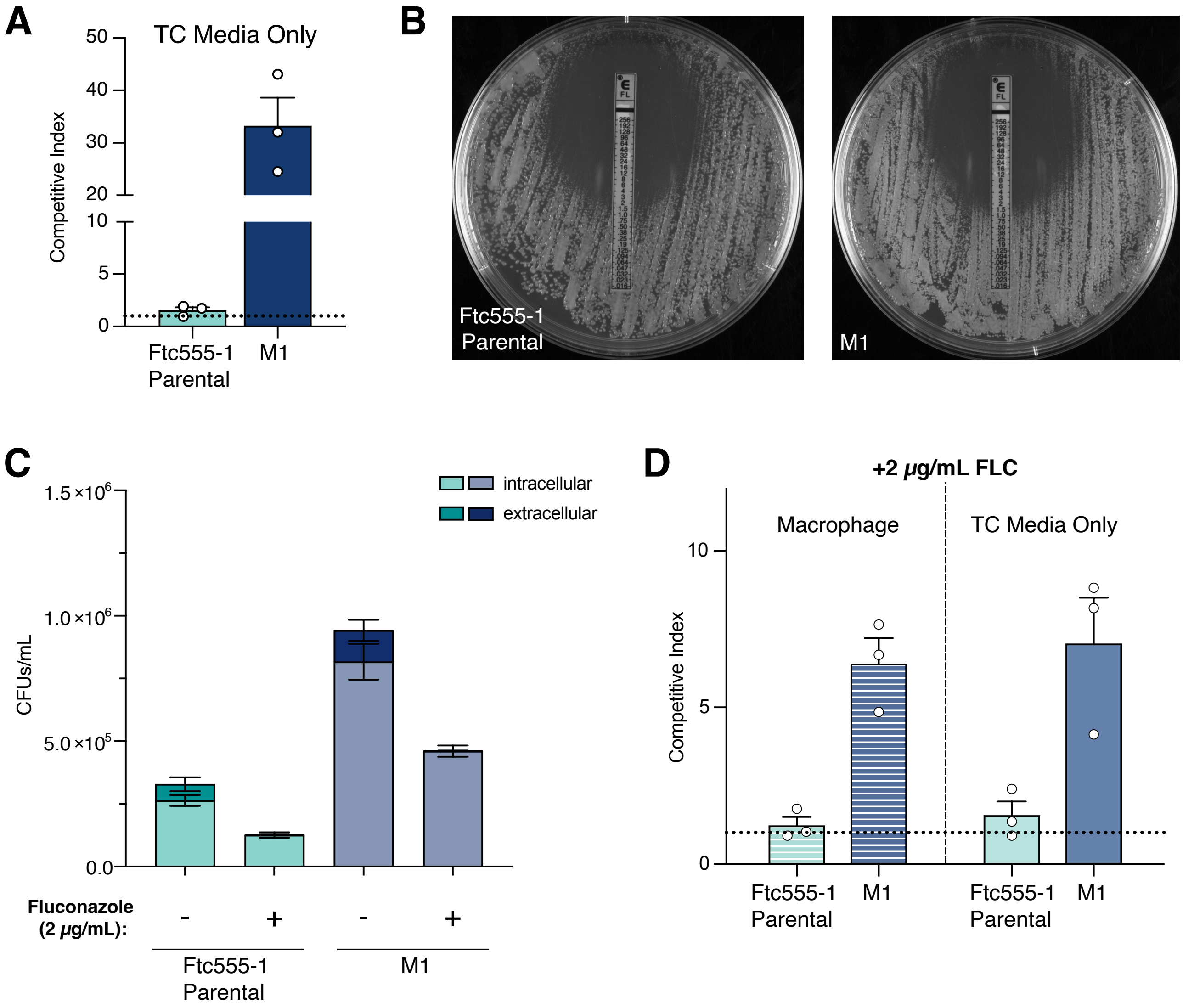

Figure S3. M1 has a fitness advantage in the macrophage intracellular niche. Related to Figure 3. (A) Competitive indices for competition experiments between the NAT^R-labeled Ftc555-1 and Ftc555-1 parental (teal) and M1 (dark blue) strains under media only conditions. The dotted line shows a competitive index of one, which correlates with no competitive advantage. (B) Minimum inhibitory concentrations (MIC) for fluconazole were determined for the Ftc555-1 parental and M1 strains using an E-test. Both strains had identical MIC values by this method of 1.5-2 μg/mL. (C) The amount of intracellular (lighter shades) and extracellular (darker shades) growth in macrophage co-incubation experiments with and without the addition of fluconazole to the culture media. Bars indicate average CFU counts ± SD from one representative experiment. (D) Competitive indices for competition experiments performed with 2 μg/mL of fluconazole added to the media after phagocytosis. Competitive index was calculated as before and data is plotted identically as in (A). Competitions were performed in macrophages (left) and in media only (right) conditions. For (A) and (D), plotted values indicate the average value ± SEM of three replicate experiments carried out on three separate days; dots indicate the average value of replicates from a single experiment.

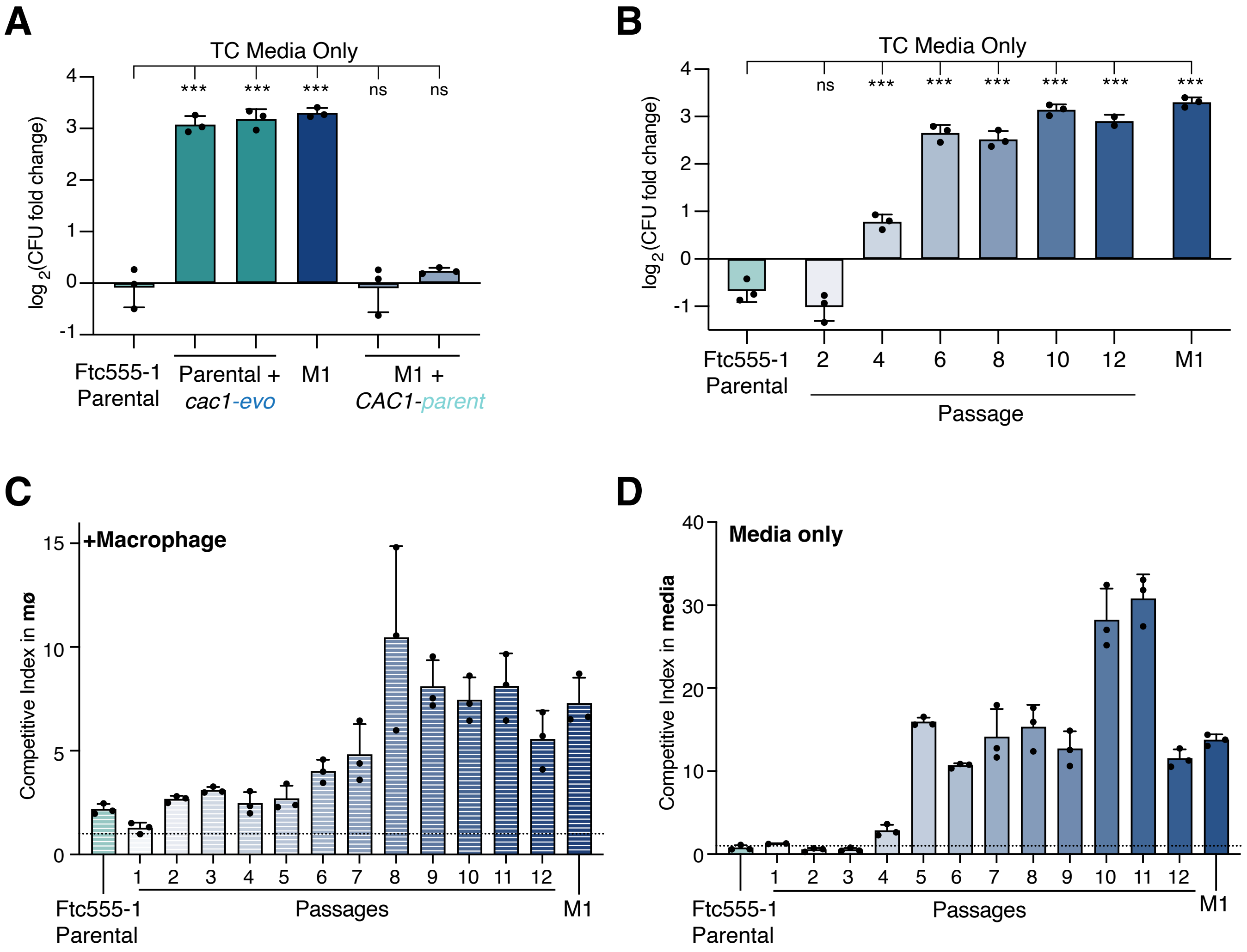

Figure S4. The M1 fitness advantage emerges following the acquisition of the *cac1-evo* allele. Related to Figure 4. Media only growth of Ftc555-1 parental, M1 and allele swap strains (A) or intermediate passage populations (B) in tissue culture media over the course of 24 hours. (C and D) Competition experiments between populations of cells from each passage and the NAT^{R}-labeled parental strain. Competitions were performed in macrophages (C) or in media-only (D). Competitive indices were calculated as before and the dotted line indicates a competitive index of one. For all panels, plotted data indicate the average values ± SD from one representative experiment. Each dot represents one replicate value from that experiment. Significance was assessed by comparison to the parental strain using ordinary one-way ANOVA followed by Dunnett's multiple comparisons test. ***p<0.0001, ns, not significant.

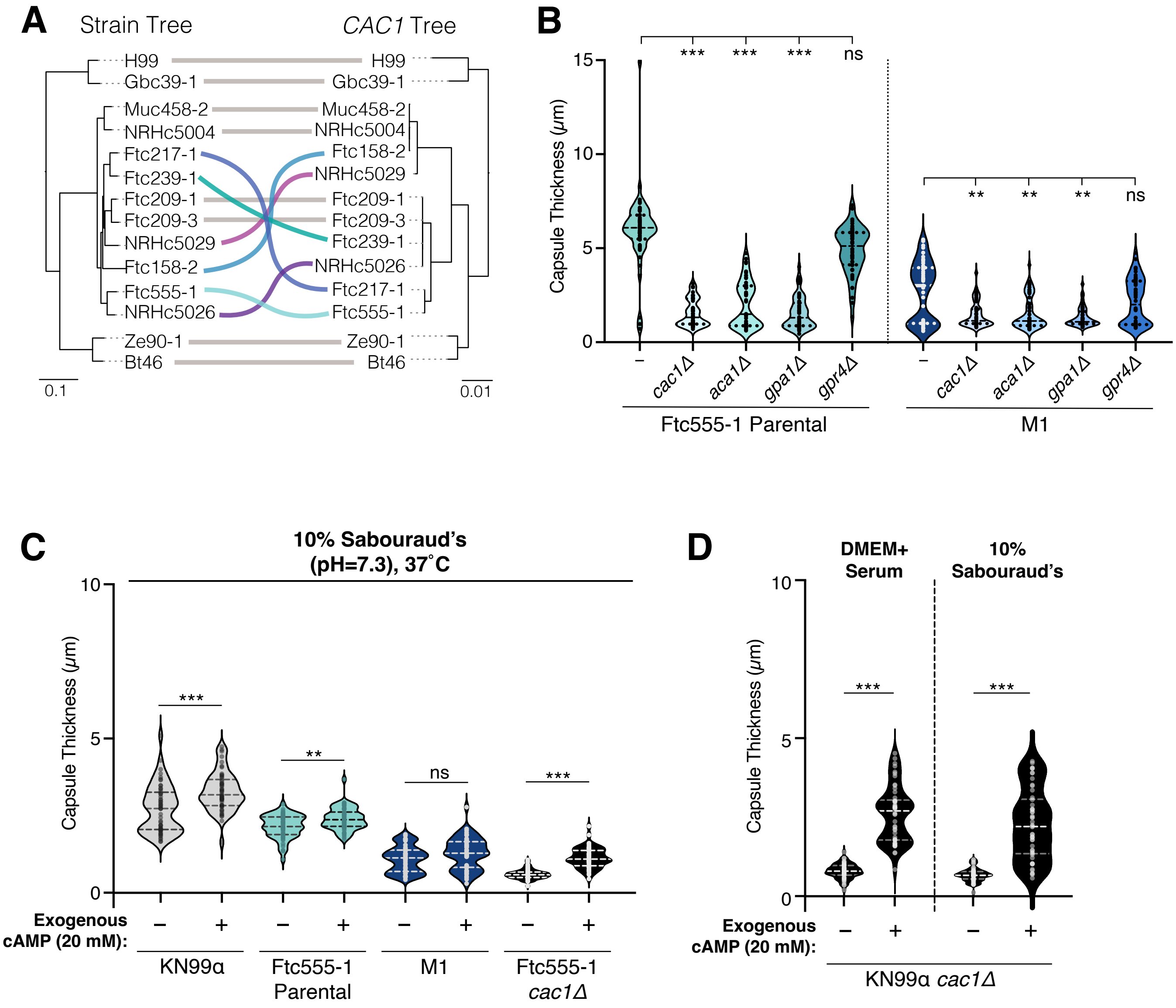

Figure S5. Differential activity of the cAMP signaling pathway in the Ftc555-1 parental and M1 backgrounds. Related to Figures 1 and 5. (A) Tanglegram comparing the strain level phylogeny (left) presented in Figure 1A to a gene tree built from the *CAC1* sequences of the strains examined in this paper (right). Concordance between the phylogenies is indicated by grey lines between strains and discordance is indicated by the colored lines showing rearrangement of the position of these strains on the phylogeny. Scale bars indicate substitutions/site. (B) Capsule size measurements in DMEM+Serum/tissue culture conditions for strains with cAMP pathway components *CAC1*, *GPA1*, *ACA1*, and *GPR4* deleted in the Ftc555-1 parental (left) and M1 (right) backgrounds. (C) Measurements of capsule sizes in 10% Sab's inducing conditions with or without the addition of 20 mM exogenous cAMP. (D) Capsule size measurements for the KN99α *cac1*Δ mutant in both tissue culture media (left) and 10% Sab's (right) in the presence and absence of exogenous cAMP (20 mM). For (B)-(D), violin plots show measurements of 50 cells per strain from one representative experiment. Significance in (B) was assessed via Kruskal-Wallis test with Dunn's multiple comparisons test. Pairwise tests of significance in both (C) and (D) were determined by Mann Whitney test. ** $p<0.01$, *** $p< 0.0001$, ns not significant

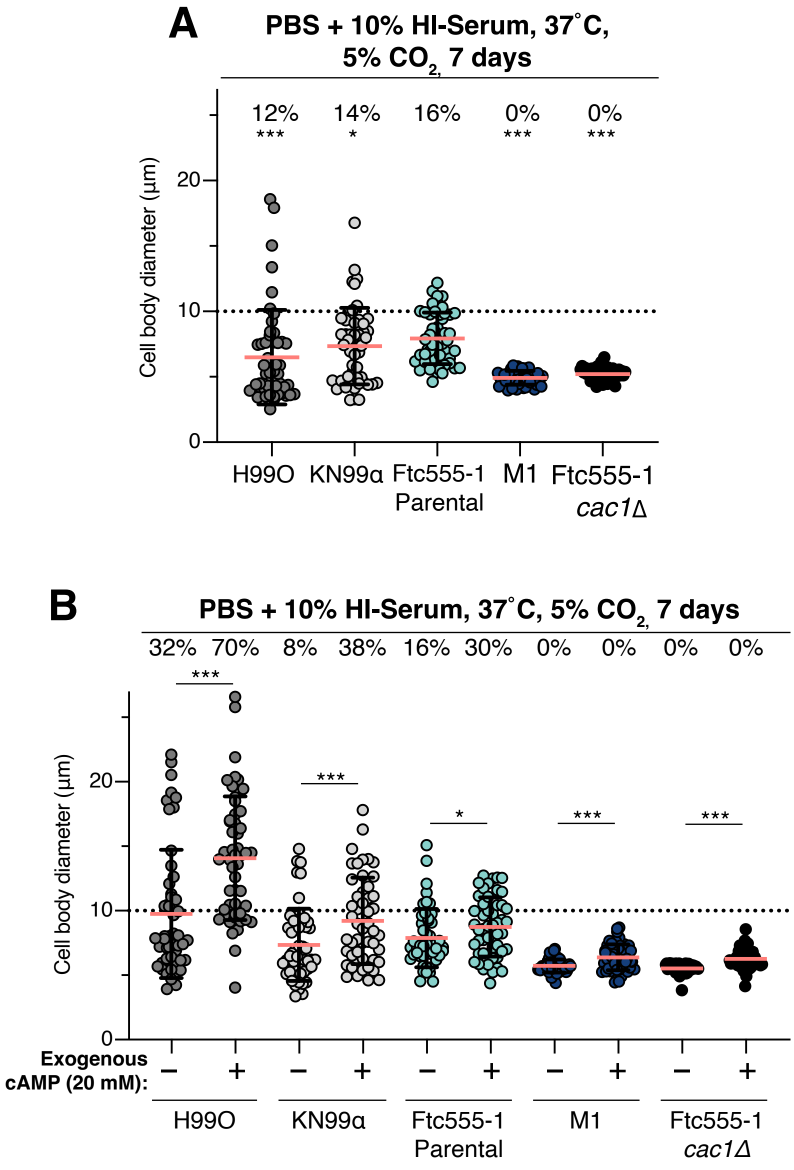

Figure S6. The *CAC1* R1227P mutation in the M1 strain affects titanization. Related to Figure 6. Measurements of cell body diameter from cells grown under an alternative titan cell induction protocol (PBS+HI-Serum for 7 days) either without cAMP supplementation (A) or comparing the effects of addition of 20 mM cAMP to the inducing media (B). 50 cells were measured per strain. Percentages above each strain indicates the percent of cells with a diameter >10 µm (indicated by the dotted line). Error bars indicate mean ± SD for one representative experiment. Significance in (A) was assessed compared to the Ftc555-1 parental strain via Kruskal-Wallis test with Dunn's multiple comparisons test. Pairwise assessment of significance between matched samples in (B) was determined by Mann Whitney test. * $p<0.05$, *** $p<0.0001$, ns not significant.

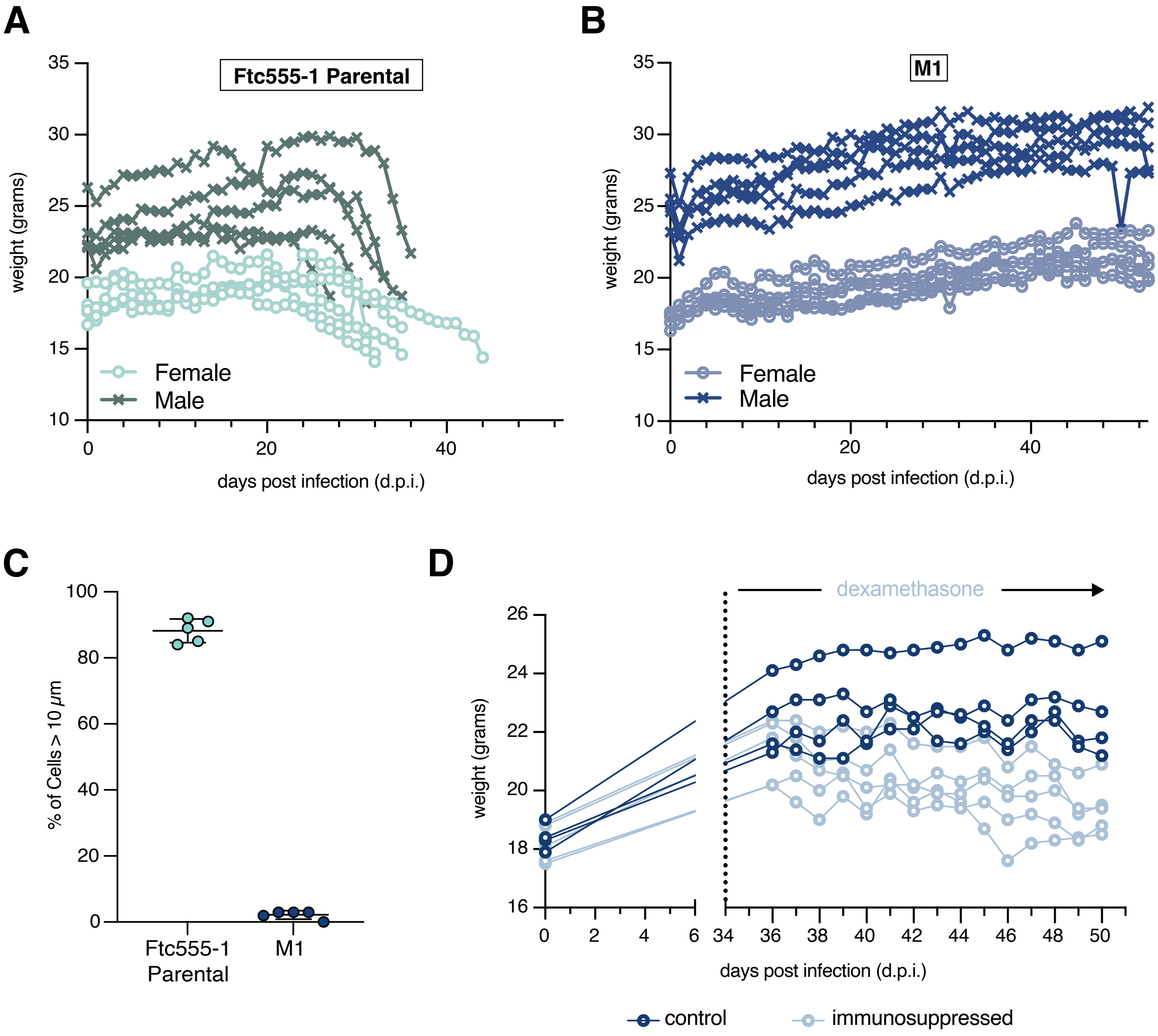

Figure S7. The M1 strain fails to cause symptomatic disease in multiple infection models. Related to Figure 7. Daily weight measurements of mice intranasally inoculated with the Ftc555-1 parental (A) or evolved M1 (B) strain. 10 animals were infected per group: five female (lighter shades) and five male (darker shades). Animals were humanely euthanized when they reached 85% of their initial mass. (C) The percentage of measured cells with a diameter greater than 10 µm from H&E stained slides was determined for each of five animals infected with the Ftc555-1 (teal) or M1 (blue) strains. The same cell measurement data from Figure 7D was used but separated by each individual infected animal. Each dot represents an individual animal. Line and error bars indicates average value ± SD. (D) Daily weight measurements of mice intranasally inoculated with the M1 evolved strain and subjected to either mock (PBS, dark blue) or dexamethasone (light blue) injections at 36 dpi to assess the role of immune suppression in controlling infection by this strain. No animals reached pre-determined clinical endpoints and the experiment was terminated after 14 days of immune suppression.

**Oligos Used for CRISPR gRNA**			
**Primer Name**	**Shorthand**	**Primer Sequence (5'>3')**	**Notes**
PCnU6_sgRNA_F	ZAH_F10	AATTGGAGCTCCACCGCG	
sgRNA_cassette_R	ZAH_F11	GGGAACAAAAGCTGGGTACC	
VNB_SH2_sgRNAcassette_F	ZAH_G26	gaatcttaggttgtggagtctGTTTTAGAGCTAGAAATAGCAAGTT	
VNB_SH2_CnPU6_R	ZAH_G27	agactccacaacctaagattcAACAGTATACCCTGCCGGTG	
CAC1AS_E0gRNA_F	ZAH_H06	gacggagaattgccgaacatGTTTTAGAGCTAGAAATAGCAAGTT	
CAC1AS_E0gRNA_R	ZAH_H07	atgttcggcaattctccgtcAACAGTATACCCTGCCGGTG	
CAC1AS_M1gRNA_F	ZAH_H08	gttgtgagggacggagaattgGTTTTAGAGCTAGAAATAGCAAGTT	
CAC1AS_M1gRNA_R	ZAH_H09	caattctccgtccctcacaacAACAGTATACCCTGCCGGTG	
CAC1KO_5'gRNA_F	ZAH_H12	gcatgggcatcctgatggcgcGTTTTAGAGCTAGAAATAGCAAGTT	
CAC1KO_5'gRNA_R	ZAH_H13	gcgccatcaggatgcccatgcAACAGTATACCCTGCCGGTG	
CAC1KO_3'gRNA_F	ZAH_H14	gtttcgtggccgactccaaagGTTTTAGAGCTAGAAATAGCAAGTT	
CAC1KO_3'gRNA_R	ZAH_H15	ctttggagtcggccacgaaacAACAGTATACCCTGCCGGTG	
GPA1KO_5'gRNA_F	MS_C12	gaagatgtggagcgatgacaaGTTTTAGAGCTAGAAATAGCAAGTT	
GPA1KO_5'gRNA_R	MS_C13	ttgtcatcgctccacatcttcAACAGTATACCCTGCCGGTG	
GPA1KO_3'gRNA_F	MS_C16	gagtcacgtaaagcgttctggGTTTTAGAGCTAGAAATAGCAAGTT	
GPA1KO_3'gRNA_R	MS_C17	ccagaacgctttacgtgactcAACAGTATACCCTGCCGGTG	
ACA1KO_5'gRNA_F	MS_B32	gcctgggatgtggccatgatgGTTTTAGAGCTAGAAATAGCAAGTT	
ACA1KO_5'gRNA_R	MS_B33	catcatggccacatcccaggcAACAGTATACCCTGCCGGTG	
ACA1KO_3'gRNA_F	MS_B36	gaccgagattgtagagcactcaGTTTTAGAGCTAGAAATAGCAAGTT	
ACA1KO_3'gRNA_R	MS_B37	tgagtgctctacaatctcggtcAACAGTATACCCTGCCGGTG	
GPR4KO_5'gRNA_F	MS_C32	gagtctgttgactcggatagGTTTTAGAGCTAGAAATAGCAAGTT	
GPR4KO_5'gRNA_R	MS_C33	ctatccgagtcaacagactcAACAGTATACCCTGCCGGTG	
GPR4KO_3'gRNA_F	MS_C36	gaaagagctgaaatacattcaGTTTTAGAGCTAGAAATAGCAAGTT	
GPR4KO_3'gRNA_R	MS_C37	tgaatgtatttcagctctttcAACAGTATACCCTGCCGGTG	
**For CRISPR Repair Templates**			
SH2_5'flank_F	ZAH_F12	agtgaattcgagctcggtacccgggg*gatcc*CTCTGAGTGTAAGGTAGAAGGTTTGTATATG	for generating SH2 repair plasmid
SH2_5'flank_R	ZAH_F13	acaatagcgagtGTGATTCCCCGCGACCGACAG	for generating SH2 repair plasmid
ACT1_prom_F	ZAH_F14	cgcggggaatcacACTCGCTATTGTCCAGGCTG	for generating SH2 repair plasmid
ACT1_prom_R	ZAH_F15	ggcggccgccatCATAGACATGTTGGGCGAGTTTAC	for generating SH2 repair plasmid
NrsR_F	ZAH_F16	caacatgtctatgATGGCGGCCGCCACTCTTGAC	for generating SH2 repair plasmid
NrsR_R	ZAH_F17	ccttcacgaattcTTAGGGGCAGGGCATGCTCATG	for generating SH2 repair plasmid
TRP1_term_F	ZAH_F18	ccctgcccctaaGAATTCGTGAAGGCGGTAAG	for generating SH2 repair plasmid
TRP_term_R	ZAH_F19	ccctgagatgtcAAGCTTATAGAAGAGATGTAGAAAC	for generating SH2 repair plasmid
SH2_3'flank_F	ZAH_F20	cttctataagcttGACATCTCAGGGAGGCAGAAAG	for generating SH2 repair plasmid
SH2_3'flank_R	ZAH_F21	aacagctatgaccatgattacgcca*agctt*CGTGAATGACAATTGCGAAAC	for generating SH2 repair plasmid
SH2_homF_full	ZAH_F30	CTCTGAGTGTAAGGTAGAAGGTTTGTATATG	for amplifying SH2 repair construct with 1 kb of homology arms
SH2_homR_full	ZAH_F31	GCTTCGTGAATGACAATTGCGAAAC	for amplifying SH2 repair construct with 1 kb of homology arms
CAC1_5'Homology_F	ZAH_H38	agtgaattcgagctcggtacccgggg*gatcc*TGAAAACGCCGTCTCGCAAC	for generating CAC1 repair plasmid
CAC1_5'Homology_R	ZAH_H39	aatcctgcatgcCGCGGAAGTCAAACCATCG	for generating CAC1 repair plasmid
DrugCassette_F_w/CAC1_OH	ZAH_H40	tttgacttccgcgGCATGCAGGATTCGAGTG	for generating CAC1 repair plasmid
DrugCassette_R_w/CAC1_OH	ZAH_I01	cagttcttaaaaaGCCATGAAGATCCTGAGG	for generating CAC1 repair plasmid
CAC1_3'Homology_F	ZAH_I02	gatcttcatggcTTTTTAAGAACTGTTGTTTTGATTTG	for generating CAC1 repair plasmid
CAC1_3'Homology_R	ZAH_I03	aacagctatgaccatgattacgcca*agctt*GGGATATGGCGCATTAGG	for generating CAC1 repair plasmid
Full_CAC1_KO_RepairF	ZAH_I04	CCGTCTCGCAACTTATTCCA	for amplifying CAC1 repair template for KO with 1 kb homology arms
Full_CAC1_KO_RepairR	ZAH_I05	TGGGATATGGCGCATTAGGAT	for amplifying CAC1 repair template for KO with 1 kb homology arms
CAC1__AlleleSwap_repairtemplate_F	ZAH_H04	CGTATGCTTCTCCCATCTGAG	for amplifying CAC1 repair template from gDNA for allele swaps
CAC1_AlleleSwap_repairtemplate_R	ZAH_H05	CCGGACAAGTTGAGATAACGC	for amplifying CAC1 repair template from gDNA for allele swaps
GPA1 5' homology_fwd	MS_C06	cggccagtgaattcgagctcggtacccgggCGGCACGAACAACAGGCG	for generating GPA1 repair plasmid
GPA1 5' homology_rev	MS_C07	tgcacaccatgccactcgaatcctgcatgcCGTGAATTGGATTGAAGGCAG	for generating GPA1 repair plasmid

For CRISPR Repair Templates (cont'd)			
**Primer Name**	**Shorthand**	**Primer Sequence (5'>3')**	**Notes**
GPA1 3' homology_fwd	MS_C08	acgccgttgaatcctcaggatcttcatggcTGACCCAAAATAAAAGATTAAAAGAG	for generating GPA1 repair plasmid
GPA1 3' homology_rev	MS_C09	caggaaacagctatgaccatgattacgccaGTGTCTATATTCTTTCTGCTGAC	for generating GPA1 repair plasmid
ACA1 5' homology_fwd	MS_B26	cggccagtgaattcgagctcggtacccgggACCATCGGCACCACCAGC	for generating ACA1 repair plasmid
ACA1 5' homology_rev	MS_B27	tgcacaccatgccactcgaatcctgcatgcGATGAGGTCAATGAGCAATGAGC	for generating ACA1 repair plasmid
ACA1 3' homology_fwd	MS_B28	acgccgttgaatcctcaggatcttcatggcATAGAAAGAAGAAAGAGAGTTCGGC	for generating ACA1 repair plasmid
ACA1 3' homology_rev	MS_B29	caggaaacagctatgaccatgattacgccaCTGGCTGCAGTGAGCACG	for generating ACA1 repair plasmid
GPR4 5' homology_fwd	MS_C26	cggccagtgaattcgagctcggtacccgggTTGACAGCGGCTAAATTG	for generating GPR4 repair plasmid
GPR4 5' homology_rev	MS_C27	tgcacaccatgccactcgaatcctgcatgcGCTTTAGTCAAATCCTCTTCG	for generating GPR4 repair plasmid
GPR4 3' homology_fwd	MS_C28	acgccgttgaatcctcaggatcttcatggcAATACATTCATGGGTTTCATATATAC TG	for generating GPR4 repair plasmid
GPR4 3' homology_rev	MS_C29	caggaaacagctatgaccatgattacgccaTTGAATGCTCATATGAAGAACC	for generating GPR4 repair plasmid
DrugCassette_F	ZAH_G24	GCATGCAGGATTCGAGTG	for amplifying drug cassette for gene KO repair plasmids
DrugCassette_R	ZAH_G25	GCCATGAAGATCCTGAGGATTC	for amplifying drug cassette for gene KO repair plasmids
GPA1_5'repairF	MS_C24	ACAACAGGCGAAGGAGGATG	for amplifying GPA1 repair template for KO with 1 kb homology arms
GPA1_3'repairR	MS_C25	CCTCCTTTGCTTATTCCGCC	for amplifying GPA1 repair template for KO with 1 kb homology arms
ACA1_5'repairF	MS_C04	CCCACCAATATCACGCGAC	for amplifying ACA1 repair template for KO with 1 kb homology arms
ACA1_3'repairR	MS_C05	GCACGCGCGTATTTAGAGC	for amplifying ACA1 repair template for KO with 1 kb homology arms
GPR4_5'repairF	MS_D04	GCGGCTAAATTGGCTTCTTC	for amplifying GPR4 repair template for KO with 1 kb homology arms
GPR4_3'repairR	MS_D05	CGTAAGTGGATCCTGCTACAG	for amplifying GPR4 repair template for KO with 1 kb homology arms
**Genotyping Primers**			
Sh2 check colony PCR Alt 5' F	MS_B01	TGTTTGTCGCCATTTTCTCC	for colony PCR genotyping of the SH2 locus, 5'
SH2 5' check R universal	ZAH_G40	GAGTGACGTTGCTGCTATTGT	for colony PCR genotyping of the SH2 locus, 5'
SH2+TRP1 check F	ZAH_G01	AATTCGTGAAGGCGGTAAGG	for colony PCR genotyping of the SH2 locus, 3'
SH2 check colony PCR Alt 3' 1 R	MS_B02	TTGTGCAATTCCCTTTGAGC	for colony PCR genotyping of the SH2 locus, 3'
CAC1_GT_5'F	ZAH_H20	CAAGCGGAGCAAGGAATGGTAAGAG	for colony PCR genotyping of CAC1 allele swaps, 5' genomic primer
CAC1_REF_5'_R1	ZAH_H22	GAATTGTGAGGGACGGAGAATTCCC	for colony PCR genotyping of CAC1 allele swaps, destabilized parental-specific primer (R1227 allele)
CAC1_GT_3'R	ZAH_H21	GAGGAGGCGTGGCTCATTTCG	for colony PCR genotyping of CAC1 allele swaps, 3' genomic primer
CAC1_ALT_3'_F2	ZAH_H30	CTCAGTTGGGTGCCAATGTCCC	for colony PCR genotyping of CAC1 allele swaps,destabilized M1-specific primer (P1227 allele)
CAC1_KO_5'checkF	ZAH_I06	GATGTGGCCATTCGTCGATC	for colony PCR genotyping of CAC1 KO, use with ZAH_G40
CAC1_KO_3'checkR	ZAH_I07	AGTGGTTGAATGGAGGAGGG	for colony PCR genotyping of CAC1 KO, use with ZAH_G01
Cas9_IntCheck_F	ZAH_G02	ACGACTCCCTCACCTTCAAG	to verify no CAS9 integration
Cas9_IntCheck_R	ZAH_G03	GACGTGCTTGGTGATCTGTC	to verify no CAS9 integration

Genotyping Primers (cont'd)			
**Primer Name**	**Shorthand**	**Primer Sequence (5'>3')**	**Notes**
GPA1_KO_5'checkF	MS_C22	CAACGCCTCTTGTTCCTAGC	for colony PCR genotyping of GPA1 KO, use with ZAH_G40
GPA1_KO_3'checkR	MS_C23	CACATCATACAAGCCTGCCC	for colony PCR genotyping of GPA1 KO, use with ZAH_G01
ACA1_KO_5'checkF	MS_C02	TGAGCCTTAGATTGTGCCATG	for colony PCR genotyping ofACA1 KO, use with ZAH_G40
ACA1_KO_3'checkR	MS_C03	GCTCTCGCTGATTCCATATGG	for colony PCR genotyping of ACA1 KO, use with ZAH_G01
GPR4_KO_5'checkF	MS_D02	TAACTGCCATTGGTCTGCTG	for colony PCR genotyping of GPR4 KO, use with ZAH_G40
GPR4_KO_3'checkR	MS_D03	TGATATGGTATCGAACAGGAGAC	for colony PCR genotyping of GPR4 KO, use with ZAH_G01
**Sequencing Primers**			
SH2_full_5'_checkF	ZAH_G34	CCAATCCAACAGCTCCGATC	amplifying the full SH2 genomic locus
SH2_full_3'_checkR	ZAH_G36	CCTCAGCCATCACTCATTCG	amplifying the full SH2 genomic locus
CAC1_upstream_GT_F	ZAH_H33	TGCCTCTGAACACCTCCAC	amplifying the region around the mutation in the CAC1 genomic locus
CAC1_downstream_GT_R	ZAH_H34	GTGACGTCCATGAGACCAAG	amplifying the region around the mutation in the CAC1 genomic locus
CAC1_sequencing_1	ZAH_H35	CATTGCCGACCTTGAGAGTG	for Sanger sequencing of the M1 derived mutation
New_CAC1_KO_5'F	ZAH_I16	GGAGTTTACTTATATCGCCGACA	for amplifying the full CAC1 genomic locus to confirm knockout
New_CAC1_KO_3'R	ZAH_I17	TCCGCAAAATCTTCAGGCTG	for amplifying the full CAC1 genomic locus to confirm knockout
CAC1_KO_seq_1	ZAH_I18	AAGTGTTCCAACTGTTCGCT	for Sanger sequence verification of CAC1 KO
CAC1_KO_seq_2	ZAH_I19	TTTCAAAGTGCCCTTCCGTG	for Sanger sequence verification of CAC1 KO

Table S1. Oligonucleotides used in this study. Related to STAR Methods
